# Hybrid Computational Framework Integrating Ensemble Learning, Molecular Docking, and Dynamics for Predicting Antimalarial Efficacy of Malaria Box Compounds

**DOI:** 10.3390/ijms27041875

**Published:** 2026-02-15

**Authors:** Martín Moreno, Sebastián A. Cuesta, José R. Mora, Edgar A. Márquez Brazon, José L. Paz, Guillermin Agüero-Chapin, Noel Pérez-Pérez, César R. García-Jacas

**Affiliations:** 1 Grupo de Química Computacional y Teórica (QCT-USFQ), Departamento de Ingeniería Química, Universidad San Francisco de Quito USFQ, Diego de Robles y Vía Interoceánica, Quito 170901, Ecuador; martinalejo11@hotmail.com (M.M.); sebastian.cuestahoyos@manchester.ac.uk (S.A.C.); 2 Department of Chemistry, Manchester Institute of Biotechnology, The University of Manchester, 131 Princess Street, Manchester M1 7DN, UK; 3 Grupo de Investigaciones en Química y Biología, Departamento de Química y Biología, Facultad de Ciencias Básicas, Universidad del Norte, Carrera 51B, Km 5, vía Puerto Colombia, Barranquilla 081007, Colombia; 4 Departamento Académico de Química Inorgánica, Facultad de Química e Ingeniería Química, Universidad Nacional Mayor de San Marcos, Lima 15081, Peru; jpazr@unmsm.edu.pe; 5 CIIMAR/CIMAR LA, Interdisciplinary Centre of Marine and Environmental Research, University of Porto, Terminal de Cruzeiros do Porto de Leixões, Av. General Norton de Matos s/n, 4450-208 Matosinhos, Portugal; gchapin@ciimar.up.pt; 6 Department of Biology, Faculty of Sciences, University of Porto, Rua do Campo Alegre 1021, 4169-007 Porto, Portugal; 7 Colegio de Ciencias e Ingenierías “El Politécnico”, Universidad San Francisco de Quito USFQ, Quito 170157, Ecuador; nperez@usfq.edu.ec; 8 Investigador por México, Secretaría de Ciencia, Humanidades, Tecnología e Innovación (Secihti), Ciudad de México 03940, Mexico; cesarrjacas1985@gmail.com; 9 Tecnológico Nacional de México, Instituto Tecnológico de Mérida, Unidad de Posgrado e Investigación, Av. Tecnológico, Km 4.5 S/N, Mérida 97000, Mexico

**Keywords:** drug design, malaria, predictive models, ensemble, classification, regression, molecular dynamics, machine learning

## Abstract

The emergence of drug-resistant strains of Plasmodium falciparum continues to challenge global malaria control efforts, underscoring the urgent need for novel therapeutic strategies. In this study, we present an integrative computational framework that combines ensemble machine learning, molecular docking, and molecular dynamics simulations to predict and characterize the antimalarial activity of compounds from the Malaria Box database. Initially, topographical and quantum mechanical descriptors were used to construct regression models for predicting pEC_50_ values, but due to the limited predictive performance in the global regression, a classification strategy was adopted, categorizing compounds into “active” and “very active” classes. The best ensemble classifier achieved robust performance (Acc_10_-fold = 0.738, Acc_ext_ = 0.675), with good sensitivity and specificity over individual models. Subsequent regression modeling within each class yielded high predictive accuracy, with ensemble models reaching Q^2^10-fold values of 0.810 and 0.793 for the very active and active classes, respectively. To explore potential mechanisms of action, molecular docking was performed against P. falciparum Cytochrome B, revealing strong binding affinities for most compounds, particularly those forming π–π stacking and hydrogen bonds with Glu272. Molecular dynamics simulations over 200 ns confirmed the stability of several ligand–protein complexes, including unexpected behavior from compound M31, which demonstrated stable binding despite poor docking scores, suggesting a possible competitive inhibition mechanism. Binding free energy calculations further validated these findings, highlighting several promising candidates for future experimental evaluation. This integrative approach offers a powerful platform for accelerating antimalarial drug discovery by combining predictive modeling with mechanistic insights.

## 1. Introduction

Approximately 250 million cases of malaria are reported annually, of which about 650,000 result in the death of patients [1,2]. Malaria remains a major public health challenge in endemic regions of Africa and Asia, with profound socio-economic implications [3,4]. Malaria is caused by protozoan parasites of the *Plasmodium* family found in mosquito species of the *Anopheles* family [4]. Of all the parasite species, *Plasmodium falciparum* is the main cause of fatal occurrences. In some cases, the disease can be appropriately controlled; however, in recent years, some obstacles have emerged to treat it effectively [4]. The most important barrier to pharmacotherapy is the resistance that some variants of *P. falciparum* have developed to antimalarial drugs [5,6]. The gradual emergence of this barrier has caused the drugs to treat the disease to be increasingly limited; consequently, the management of infections is deficient, and the number of deaths increases significantly [4,5].

Consequently, the development of new antimalarial drugs has become an urgent challenge for the pharmaceutical industry. However, this is a complex process that requires a considerable amount of time. One of the most crucial stages in the discovery of new drugs is the selection of compounds for in vitro and in vivo studies. Over the past decades, computational tools have been extremely useful for identifying the most promising candidates. Computer-aided drug design (CADD) encompasses a series of approaches used to discover and analyze chemical compounds exhibiting biological activity [7]. Currently, Quantitative Structure-Activity Relationship (QSAR) is one of the most widely used CADD techniques.

Many QSAR studies for antimalarial drug discovery have been reported in the last decade [3]. The increasing emergence of resistance to existing therapies and the limited availability of effective vaccines have intensified the search for new antimalarial candidates. Currently used drugs include chloroquine, artemisinin derivatives, sulfadoxine, pyrimethamine, mefloquine, primaquine and atovaquone, which were largely discovered through experimental medicinal chemistry and screening approaches (10.1080/17460441.2021.1866535). In recent years, computational methods such as QSAR, molecular docking and virtual screening have become essential tools to guide the design and optimization of new antimalarial compounds by identifying relationships between molecular structure and biological activity

In the literature, the most used approaches for the selection of molecular descriptors are: 2D-QSAR (use of 2D descriptors), 3D-QSAR (use of 3D descriptors) and 2D-3D-QSAR (mixed use of 2D and 3D descriptors) [3,8,9,10]. Most studies use linear regression techniques or non-linear ML techniques [3,11]. For the present study, 2D and 3D molecular descriptors were employed, combining a wide variety of ML techniques. This approach ensured an in-depth level of exploration during the modeling phase.

The use of ensemble models is one of the least frequently used approaches in QSAR studies against malaria. An ensemble model is an ML technique that combines the predictions of multiple individual models to improve statistical performance [12]. In the last six years, only one classification study using ensemble models has been conducted [13]. In this study, 5697 biologically active compounds against malaria were used, and the statistical performance was satisfactory. Additionally, studies belonging to other fields showed that ensemble-type models are quite promising, which is why this approach was chosen for the present work [12,14].

The selection of the database plays an important role in the quality of the results and the extension of the applicability domain [3]. The applicability domain is a theoretical region in space that is delimited by the molecular descriptors that make up the model. If the database used for modeling contains structurally similar molecules, the applicability domain is narrowed considerably as the molecular descriptors span very little domain space. In antimalarial QSAR studies, dataset size is often a limiting factor, as many published models rely on relatively small datasets ranging from tens to about one hundred compounds [13]. Therefore, the use of a dataset comprising approximately 400 compounds in the present work represents a substantially larger and more diverse chemical space, allowing a broader and more reliable applicability domain.

The database that was selected for modeling bears the name of Malaria Box, which consists of 400 chemical compounds that have biological activity against *Plasmodium falciparum* [2]. Malaria Box reports antimalarial activity using half the maximum effective concentration (EC50) [2]. The EC50 values for the 400 molecules are between 30 nM and 4 μM, which implies that the range of biological activity is wide [2]. The Malaria Box has the advantage of molecular variability, meaning that the molecules comprising it do not exhibit similar structures. This benefits modeling as the objective is to enhance the applicability domain (AD). This study aims to develop robust ensemble-based QSAR models integrating 2D, 3D, and quantum descriptors to predict antimalarial activity of Malaria Box compounds, complemented by molecular docking and dynamics simulations.

## 2. Results

### 2.1. Regression Models

As an initial approach to building a predictive model, feature selection for regression was performed using the 317 molecules in the database. The search for the best descriptors was conducted separately for 2Dt-QM and 3Dt-QM. Each subset of descriptors was labelled with its respective regression technique, search method, and the number of molecular descriptors. The first statistical parameter used for the selection of the best models was the Q^2^_10-fold_, followed by the MAE_10-fold_. According to the theoretical framework, the best models have Q^2^_10-fold_ values close to 1 and MAE_10-fold_ values near 0 [15].

A total of 29 models were built using Weka 3.8, with their statistics provided in Table A1. Table 1 summarizes the five best models for both 2Dt-QM and 3Dt-QM. It is important to note that these subsets were obtained after an initial search. As observed, the models built with 2Dt-QM do not exceed 0.4 for their Q^2^_10-fold_, while there are two interesting models for 3Dt-QM with a Q^2^_10-fold_ less than 0.8. However, given that the Q^2^_10-fold_ barely reached 0.792 with 144 molecular descriptors, reducing the number of descriptors is not a viable path, as it automatically lowers the Q^2^_10-fold_ below 0.5. In 100% of the cases, a second feature selection with a significant reduction (10 or more descriptors) greatly disturbs the statistical parameters of the model. Therefore, the study was approached from a different perspective due to the poor performance of the global models in terms of their statistical parameters.

### 2.2. Global Classification Models

The alternative path chosen was global classification modeling. For each cutoff, feature selection was performed separately for 2Dt-QM and 3Dt-QM, resulting in a total of 490 subsets. Models with more than 30 descriptors were discarded, yielding a total of 243 models for analysis. The statistics for these models are provided in Table A2. The main parameter considered for selecting the best models was Acc_10-fold_, followed by Sens_10-fold_ and Spec_10-fold_. According to the literature, the best models have largerAcc_10-fold_ values, and Sens_10-fold_ and Spec_10-fold_ close to 1. These last two parameters indicate the reliability with which the model classifies A and B, respectively.

Table 2 presents a summary of the top-performing models identified for each EC_50_ cutoff, evaluated using both 2Dt–QM and 3Dt–QM descriptor sets. Each model is annotated with its corresponding classification algorithm, search strategy, and number of selected descriptors. Overall, most models achieved acceptable predictive performance, with 10-fold cross-validated accuracy values exceeding 0.70, and models based on cutoffs above 1.0 reaching accuracy above 0.80.

However, accuracy alone was not sufficient to guide threshold selection. A detailed analysis of sensitivity and specificity revealed pronounced imbalances at the extremes of the cutoff range. Models constructed using a cutoff of 0.6 exhibited low sensitivity, indicating a poor ability to correctly identify highly active compounds. Conversely, cutoffs between 0.9 and 1.2 resulted in substantially reduced specificity, reflecting an increased rate of false positive classifications. These effects were directly associated with highly skewed class distributions at those thresholds, leading to biased classifiers with limited generalization capability.

To ensure statistical robustness and balanced predictive behavior, cutoffs producing strongly imbalanced class distributions were excluded from further consideration. The thresholds of 0.7 and 0.8 were therefore retained, as they provided a more equitable distribution of compounds between classes and yielded models with a better balance between sensitivity and specificity. This selection minimizes classification bias while maintaining biological relevance, corresponding to EC_50_ values.

When comparing the best models of both cutoffs, it is observed that both exhibit similar statistics for 2Dt-QM, with negligible differences in their sensitivity and specificity values. However, in general, the 3Dt-QM models for the 0.7 cutoff show better Acc_10-fold_ than the models with the 0.8 cutoff. This trend is more evident in Table A2. Therefore, 0.7 was chosen as the best cutoff for classification modeling, and 3Dt-QM as the best type of descriptors for modeling. In total, nine 3Dt-QM classification models proceeded to the next stage, where the best was RF_BF_12 (M1_CLASS).

The next step was partitioning into training/test sets. Clustering was performed using the k-means technique, resulting in 13 clusters of molecules based on similarity. Within each cluster, 75% of the molecules were randomly selected to form the training set and the remaining 25% to form the test set. Once the files for the nine models were created, an applicability domain analysis was conducted using AMBIT Discovery. The results were satisfactory, as the training sets had 100% coverage for the test sets. Finally, the performance and robustness of the classification models were evaluated using the following tests: 10-fold cross-validation for the training set, test set prediction (Ext), and leave-one-out cross-validation (LOO). Table A3 compiles the statistics for the validation of the nine best global classification models.

**Figure A1:**
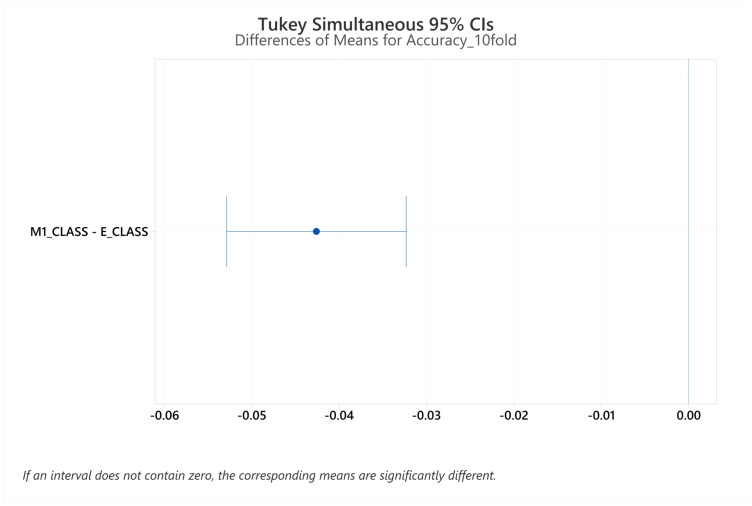
Tukey analysis for the accuracy (Acc 10-fold) of the best individual classification model (M1_CLASS) and the ensemble model (E_CLASS) with 95% CIs.

**Figure A2:**
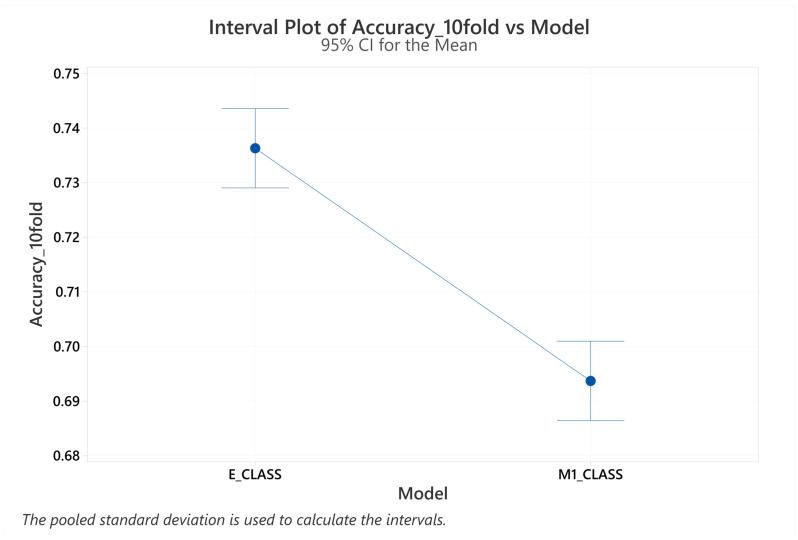
Interval plot for the accuracy (Acc 10-fold) of the best individual classification model (M1_CLASS) and the ensemble model (E_CLASS) with 95% CIs.

**Figure A3:**
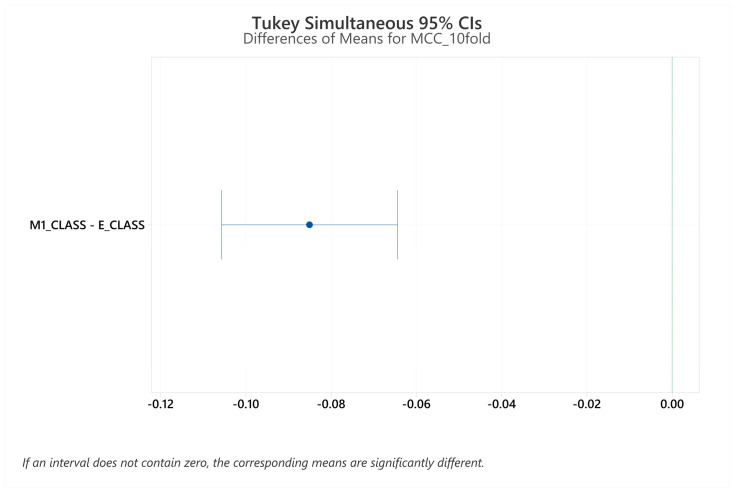
Tukey analysis for the MCC 10-fold of the best individual classification model (M1_CLASS) and the ensemble model (E_CLASS) with 95% Cis.

**Figure A4:**
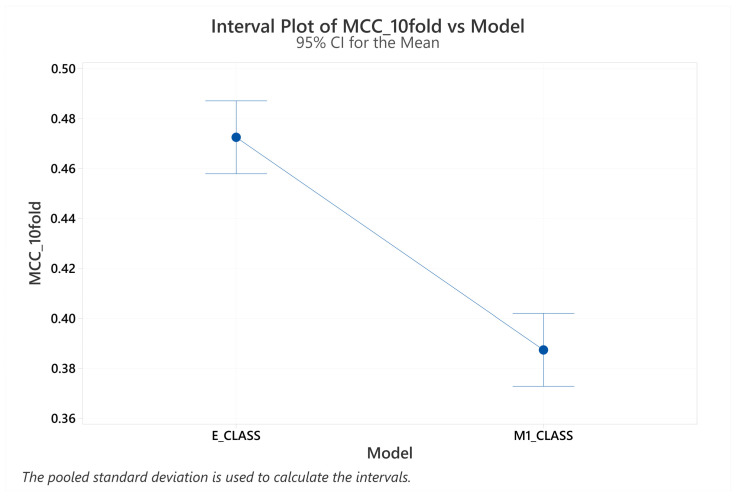
Interval plot for the accuracy (Acc 10-fold) of the best individual classification model (M1_CLASS) and the ensemble model (E_CLASS) with 95% CIs.

To continue with the classification modeling, an ensemble model was built from four models in Table A3 (J48_GS_12, IBK_IWSS_8, RF_IWSS_12, RF_BF_12), and its performance was evaluated with the validation tests described above. Table 3 summarizes the statistical parameters for the best individual classification model and the ensemble model. It is observed that E_CLASS outperforms M1_CLASS in most statistical parameters. This occurs because E_CLASS was built with four individual models, significantly reducing the error in classifying a subset of molecules. Lastly, an ANOVA analysis was conducted for Acc_10-fold_ and MCC_10-fold_ using a 95% significance level. Ten observations of these parameters were calculated for each model using a random seed in the cross-validation, finding that M1_CLASS and E_CLASS are statistically different. ANOVA results are shown in Table A4 and Figure A1, Figure A2, Figure A3 and Figure A4.

**Figure 1:**
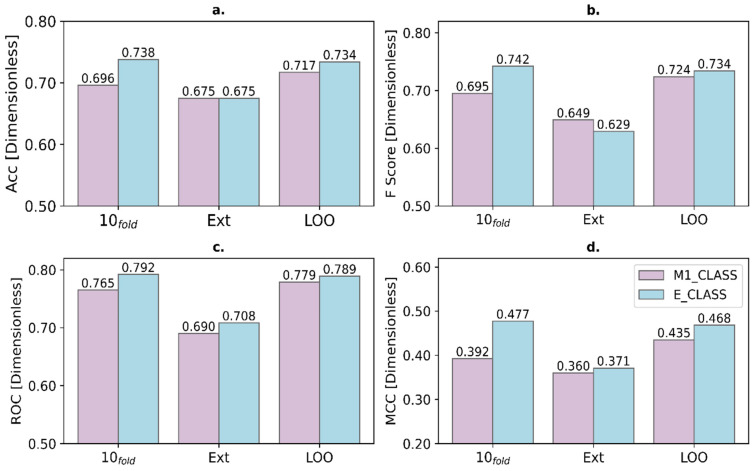
Comparison between the best individual classification model (M1_CLASS) and the ensemble model (E_CLASS) for accuracy (**a**), F-score (**b**), area under the ROC curve (**c**), and Matthews correlation coefficient (**d**).

Additionally, a detailed comparison was made of four statistical parameters commonly used in the literature to analyze the robustness of binary classification models (accuracy, F-score, area under the ROC curve, and Matthews correlation coefficient). These four parameters together provide a good estimate of model performance, class balance, and prediction reliability [15,16]. A reliable statistical model has values for these parameters close to 1. As shown in Figure 1, E_CLASS outperforms or matches M1_CLASS in performance and reliability across the three validation tests. This demonstrates that the use of ensemble models significantly improves the performance of a predictive classification model.

Finally, the molecular descriptors of the models used to construct E_CLASS were analyzed. Among the four models, including M1_CLASS, at least 60% of the topographic descriptors were calculated based on three chemical properties: polarizability, charge, and the octanol/water partition coefficient. This suggests that the distribution of the electron cloud, electrostatic interactions, and behavior in an aqueous medium significantly influence the distinction between molecules with higher and lower biological activity.

### 2.3. Construction and Validation for Each Class Regression Models

After constructing the global classification model, the next phase was a separate regression modeling. The procedure was the same for both classes. First, feature selection for regression was performed for the very active class (A) and the active class (B). Only 3Dt-QM descriptors were used due to the poor statistical performance of the 2Dt-QM descriptors. The first statistical parameter used for selecting the best models was Q^2^_10-fold_, followed by MAE_10-fold_. Initially, a total of 15 models were constructed for each class, and Table 4 summarizes the statistical parameters of the top five.

In Table 4, it is observed that the models reached Q^2^_10-fold_ very close to 1. However, the number of descriptors in these models exceeds 30 for both class A and class B. Therefore, a reduction in the number of descriptors forming the top four models for each class was performed using Weka 3.8 and QSARINS. The risk assumed in this procedure was the perturbation of Q^2^_10-fold_. performance. The statistical parameters for all models, including those from the first search, are found in Table A5. Five models were chosen for class A, with the best being GP_BF_101_QSARINS_20 (M5_REG_A), and four models for class B, with the best being GP_BF_112_LR_BF_28 (M1_REG_B). These nine models were the optimal candidates for the next phase of modeling.

For the first part of the validation, the training/test partition performed in the global classification modeling was maintained. Once the files were created, the applicability domain (AD) of the models was analyzed using AMBIT Discovery, obtaining 100% coverage of the training set for the test set. Finally, the performance and robustness of the regression models were evaluated using five statistical tests (correlation analysis, 10-fold cross-validation for the training set, leave-one-out cross-validation, test set prediction, and dependent variable randomization). The best models showed R^2^ and Q^2^ values close to 1, indicating good correlation and predictability. Furthermore, the values for mean absolute error (MAE), root mean square error (RMSE), and Q^2^_y-Scrambling_ were close to 0. This implies that the models do not predict pEC50 at random and do so with good accuracy. The statistical parameters for the individual models of class A are found in Table A6, and for class B in Table A7.

**Figure A5:**
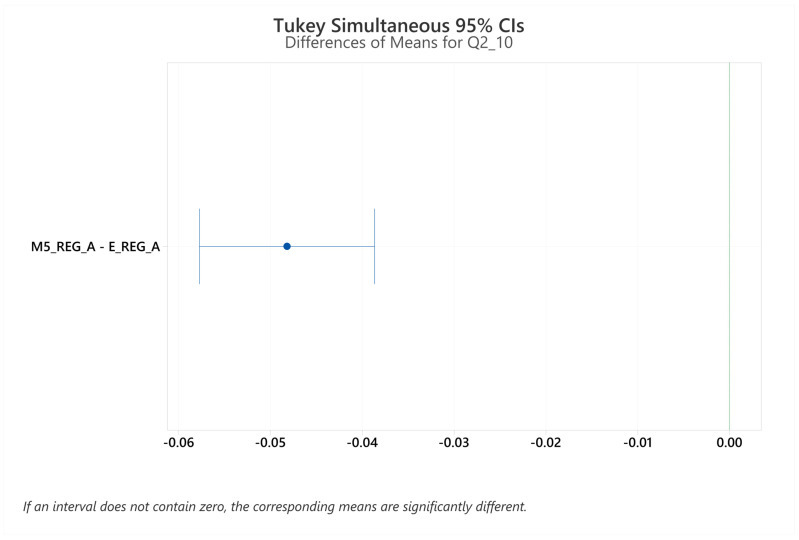
Tukey diagram for the Q^2^10-fold of the best individual regression model for class A (M5_REG_A) and the ensemble model (E_REG_A) with 95% CIs.

**Figure A6:**
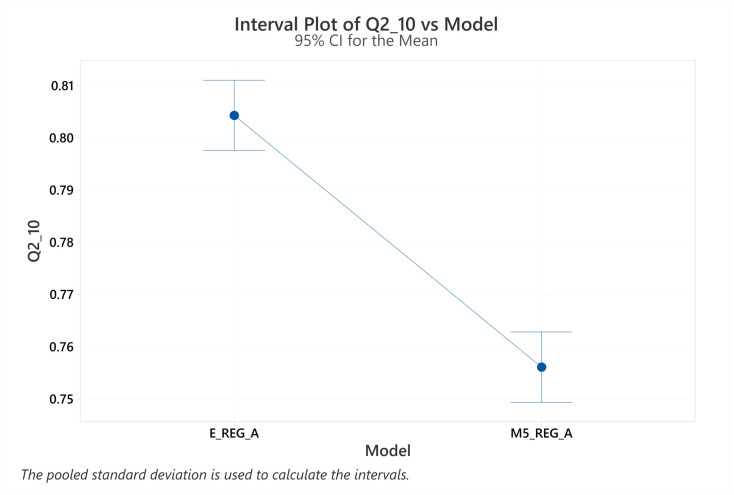
Interval plot for the Q^2^10-fold of the best individual regression model for class A (M5_REG_A) and the ensemble model (E_REG_A) with 95% Cis.

**Figure A7:**
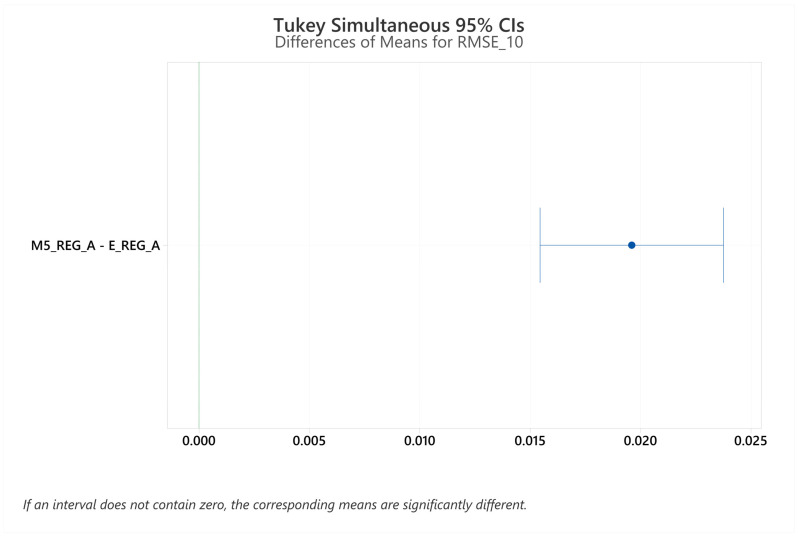
Tukey diagram for the RMSE 10-fold of the best individual regression model for class A (M5_REG_A) and the ensemble model (E_REG_A) with 95% CIs.

**Figure A8:**
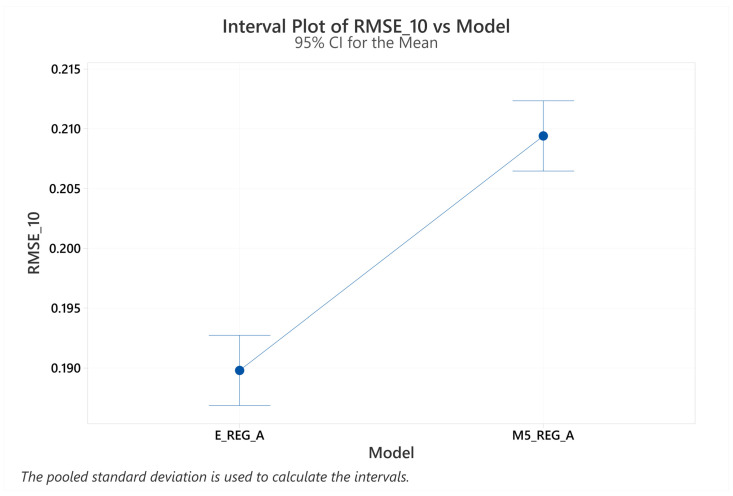
Interval plot for the RMSE 10-fold of the best individual regression model for class A (M5_REG_A) and the ensemble model (E_REG_A) with 95% CIs.

**Figure A9:**
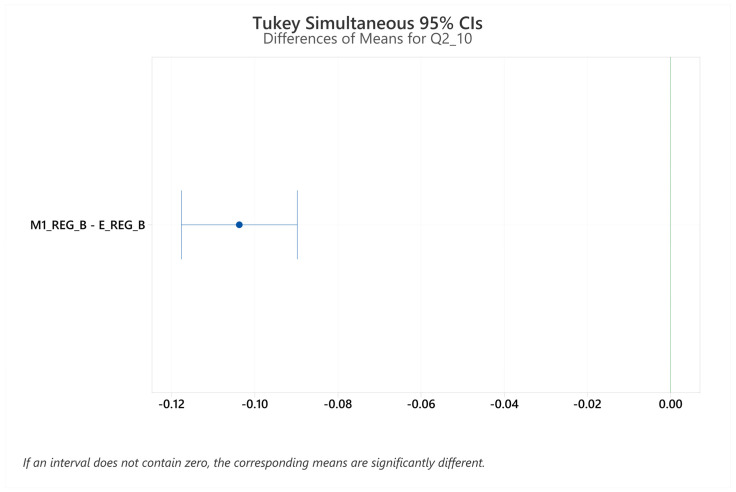
Tukey diagram for the Q^2^10-fold of the best individual regression model for class B (M1_REG_B) and the ensemble model (E_REG_B) with 95% CIs.

**Figure A10:**
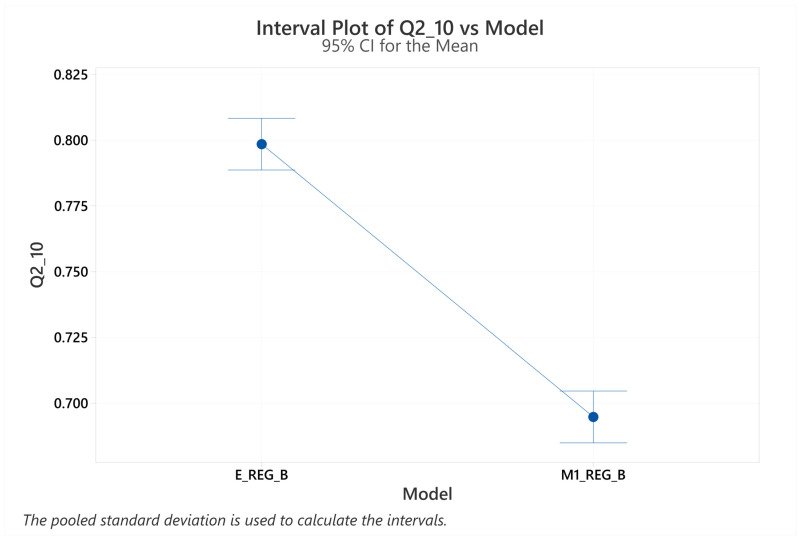
Interval plot for the Q^2^10-fold of the best individual regression model for class B (M1_REG_B) and the ensemble model (E_REG_B) with 95% CIs.

**Figure A11:**
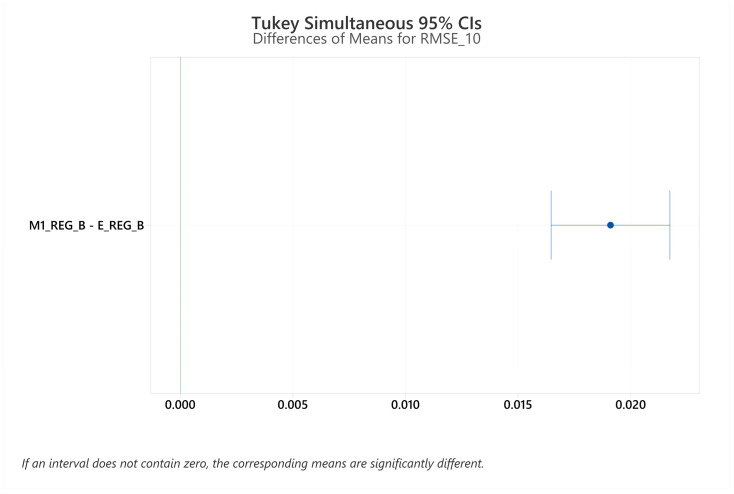
Tukey diagram for the RMSE 10-fold of the best individual regression model for class B (M1_REG_B) and the ensemble model (E_REG_B) with 95% CIs.

**Figure A12:**
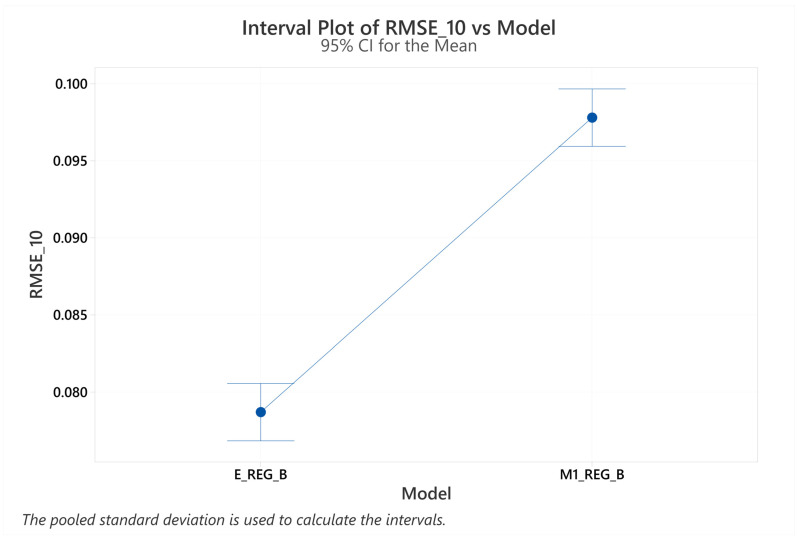
Interval plot for the RMSE 10-fold of the best individual regression model for class B (M1_REG_B) and the ensemble model (E_REG_B) with 95% CIs.

The next step was the construction of an ensemble model for each class using the models in Table A6 and Table A7. For class A, the ensemble (E_REG_A) was built using the predictions of the models: GP_BF_101_QSARINS_20, LR_GS_71_QSARINS_20, and LR_BF_70_SMOR_BF_22. For class B, the ensemble (E_REG_B) was constructed using the predictions of: LR_BF_44_QSARINS_20, GP_BF_112_QSARINS_20, and GP_BF_112_LR_BF_28. Both ensembles were evaluated with the previously described validation tests, and their performance was compared to the best individual model for each class. The statistical parameters for validation are compiled in Table 5, showing that the ensemble models outperform the individual models in all their statistics. An ANOVA analysis was performed for Q^2^_10-fold_ and RMSE_10-fold_ using a 95% significance level. Ten observations of these parameters were calculated for each model using a random seed in cross-validation, and it was found that the ensemble models (E_REG_A and E_REG_B) are statistically different from their individual models (M5_REG_A and M1_REG_B). The ANOVA results for each class A correspond to Table A8 and Figure A5, Figure A6, Figure A7 and Figure A8. The ANOVA results for class B correspond to Table A9 and Figure A9, Figure A10, Figure A11 and Figure A12.

**Figure 2:**
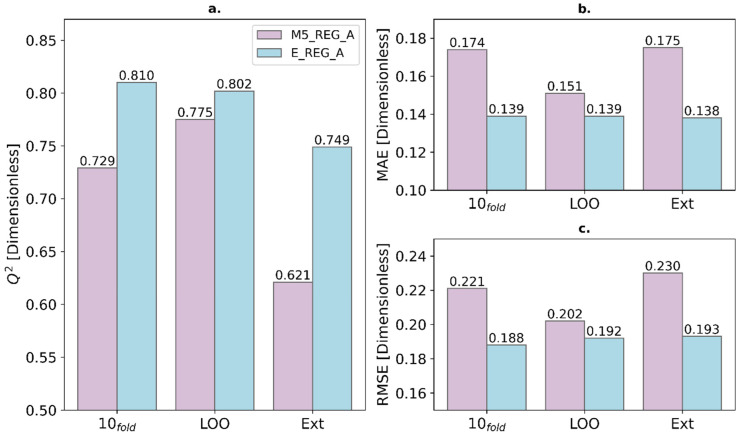
Comparison for the Very Active class (A) between the best individual regression model (M5_REG_A) and the ensemble model (E_REG_A) for its Q2 (**a**), mean absolute error (**b**), and root mean square error (**c**).

**Figure 3:**
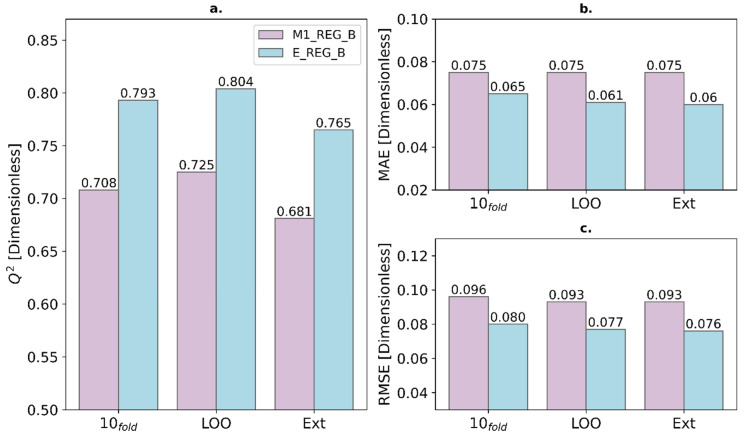
Comparison for the Active class (B) between the best individual regression model (M1_REG_B) and the ensemble model (E_REG_B) for its Q^2^ (**a**), mean absolute error (**b**), and root mean square error (**c**).

Additionally, a detailed comparison of Q^2^, MAE, and RMSE was performed for three validation tests. In Figure 2 and Figure 3, the ensemble models significantly outperform the best individual models in their performance. The most significant differences are found in the 10-fold cross-validation and the prediction of the test set. This means that the predictions obtained with the ensemble model are much more reliable. For this reason, one of the projects with the greatest long-term impact is the implementation of the models in this study for screening external databases to find new candidates against malaria.

A more detailed analysis of the molecular descriptors that participated in the ensemble models was carried out. For both class A and class B, it was observed that at least 60% of the descriptors were calculated based on four chemical properties: polar surface area, electronegativity, octanol/water partition coefficient, and van der Waals volume. These properties are not only statistically relevant for distinguishing compounds with higher antiplasmodial activity, but they also play a mechanistic role in ligand–target interactions, particularly with Cytochrome BC1. For example, Polarizability, which reflects the ability of a molecule’s electron cloud to deform in response to external fields, has been shown to significantly influence binding affinity in biological systems. It affects how well a ligand can adapt to the electrostatic environment of a protein’s active site, enhancing van der Waals and dispersion interactions [17]. In the context of Cytochrome BC1, which contains a hydrophobic binding pocket and charged residues such as Glu272, polarizable ligands are better suited to form stable, non-covalent interactions.

Electrostatic charge is equally critical, as it governs the formation of hydrogen bonds and ionic interactions with charged amino acid residues in the binding site. For Cytochrome BC1, electrostatic complementarity between the ligand and residues like Glu272 and Tyr279 can stabilize the complex and improve inhibitory potency [18].

LogP, a measure of lipophilicity, influences the ligand’s ability to partition into the hydrophobic environment of the mitochondrial membrane and the binding pocket of Cytochrome BC1. Compounds with optimal logP values are more likely to reach and remain within the target site, enhancing bioavailability and binding efficiency [19].

Together, these descriptors provide not only predictive power in QSAR modeling but also mechanistic insight into how molecular properties translate into effective inhibition of Cytochrome BC1. Therefore, these are factors to be considered in the search and development of new drugs against *P. falciparum*.

### 2.4. Molecular Docking and Molecular Dynamics

**Figure 4:**
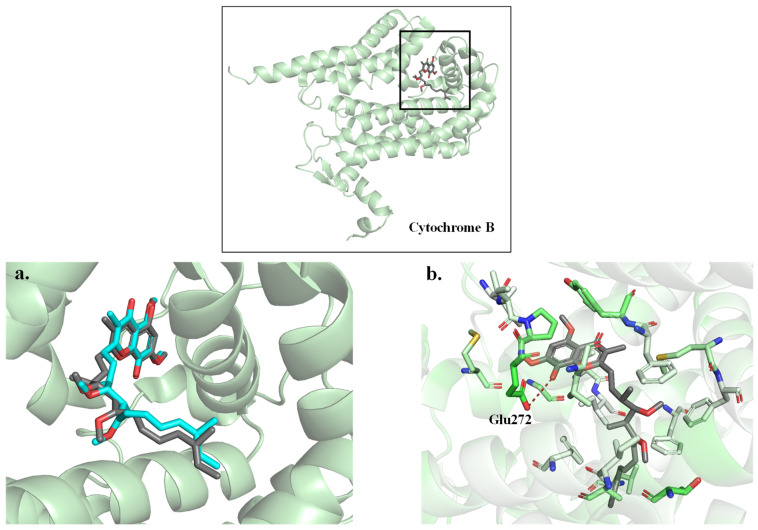
Cytochrome B–Stigmatellin A complex. The protein is shown in light green, the experimental ligand in grey and the docked ligand in cyan (**a**). Amino acids in the active site are presented in sticks and coloured based on the Eisenberg hydrophobicity scale from white (hydrophobic) to green (hydrophilic) (**b**).

The first step in the computational pipeline involved establishing and validating a robust docking protocol. To achieve this, a methodology validation was conducted using the co-crystallised ligand Stigmatellin A, which binds to the Cytochrome B subunit and redocks it to the protein. The resulting docking pose with the lowest energy showed a nearly identical pose compared to the electron density of the ligand in the solved structure (Figure 4a). This strong overlap confirmed the robustness and suitability of applying the selected docking protocol in this system. Cytochrome B-Stigmatellin A complex, the ligand occupies a predominantly hydrophobic cavity within the receptor, with Glu272 being the only polar residue present in the binding site. This observation suggests that hydrophobic interactions are likely to play a dominant role in stabilising ligand binding. However, the hydroxyl group in Stigmatellin A, which is located within its hydroxychromone moiety, forms a hydrogen bond with Glu272. This interaction could be essential for enhancing its inhibitory activity (Figure 4b). Therefore, to design or select future ligands to serve as lead compounds or inhibitors, the ability to form a hydrogen bond (HB) with Glu272 may serve as a useful criterion to improve potency.

Upon successful validation of the docking protocol, all 317 compounds from the Malaria Box database were subjected to molecular docking simulations against the Cytochrome B subunit of the Cytochrome bc1 complex. The results revealed that 308 of these compounds exhibited favorable binding affinities, with docking scores ranging from −11.8 to −6.1 kcal/mol (Table A10), indicative of strong interactions with the target site.

A smaller subset of eight molecules (M54, M307, M319, M342, M346, M370, M371, and M37) showed moderately negative scores between −5.9 and −3.1 kcal/mol, suggesting weaker binding or partial incompatibility with the active site geometry. Notably, two compounds (M27 and M31) yielded positive docking scores, implying unfavorable interactions and poor fit within the binding pocket. These findings suggest that such molecules may exert their antiplasmodial effects through alternative mechanisms unrelated to Cytochrome BC1 inhibition.

**Figure 5:**
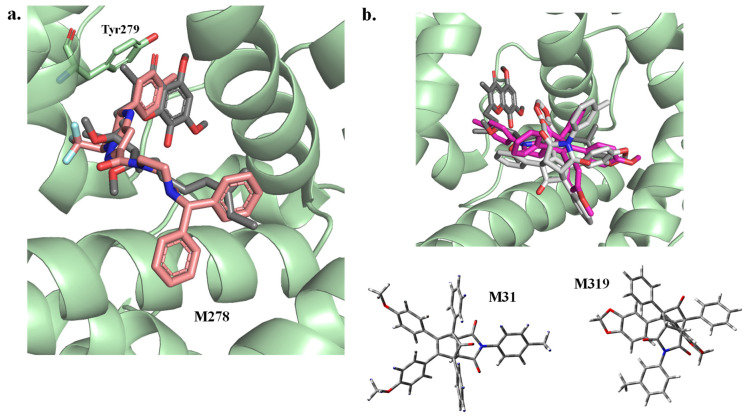
Comparative Docking Poses of Representative Malaria Box Ligands (M278, M31, and M319) within the Cytochrome B Binding Site (**a**). M278 is shown in salmon, M31 in purple, and M319 in white (**b**).

**Figure A13:**
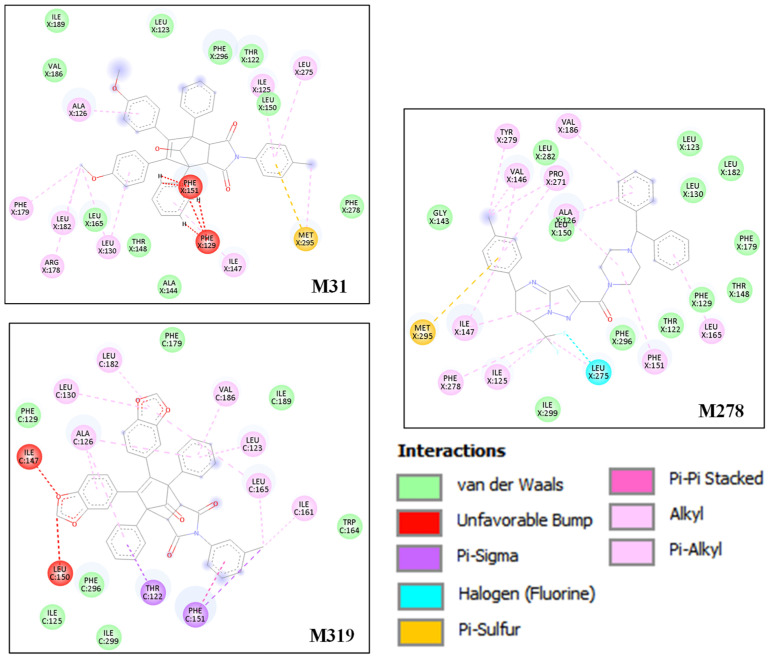
Two-dimensional diagram of the docking poses for compounds M31, M278, and M319.

To gain deeper insight into binding behavior, three representative compounds were selected for detailed structural analysis (Figure A13): M278 (strong binder, −11.8 kcal/mol), M319 (moderate binder, −3.1 kcal/mol), and M31 (poor binder, +3.6 kcal/mol). The docking pose of M278 revealed an elongated conformation that closely mimicked the binding orientation of Stigmatellin A, a known Cytochrome BC1 inhibitor. Remarkably, the phenyl ring of M278 overlapped spatially with the hydroxychromone moiety of Stigmatellin A, suggesting a potential π–π stacking interaction with Tyr279 (Figure 5a), a feature known to enhance molecular recognition and binding stability. In contrast, M319 and M31 displayed bulky, three-dimensional scaffolds that were poorly accommodated within the narrow binding cavity of Cytochrome B. This steric hindrance likely contributed to their suboptimal docking scores and limited interaction with key residues (Figure 5b). These observations support the hypothesis that such compounds are unlikely to act as direct inhibitors of Cytochrome BC1 and may instead target other biochemical pathways within the parasite.

To complement the docking study and assess the temporal stability of these interactions, molecular dynamics (MD) simulations were conducted on a selected subset of 20 ligands. These included 10 compounds randomly selected from the active group and 10 from the most active subset. M31 was specifically chosen to act as a negative control due to its poor docking score, while Stigmatellin A served as a positive control given its established activity against Cytochrome B.

For each ligand, the pose with the lowest docking score was used as the starting conformation for the MD simulation. The reliability of each simulation was assessed by monitoring system properties such as density, box size, volume, pressure, and energy fluctuations. The dynamics of the protein–ligand complexes were evaluated using root-mean-square deviation RMSD plots, analyzing hydrogen bond formation, and visualizing the structural changes throughout the simulation. The binding free energy was also estimated to complement the structural analyses.

**Figure 6:**
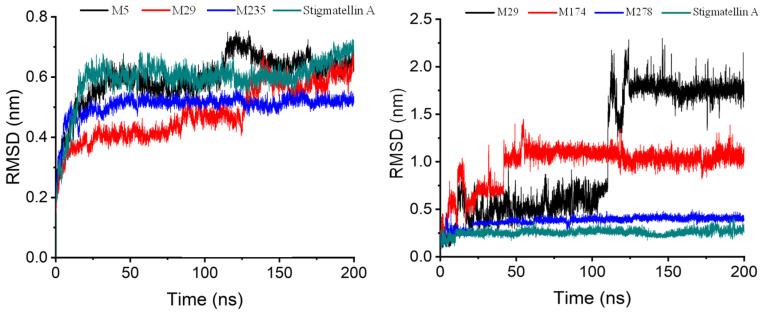
RMSD of Cytochrome B in Stigmatellin A, M5, M29, and M235 systems (**left**), and M29, M174, and M278 systems (**right**) during the 200-ns simulation.

**Figure A14:**
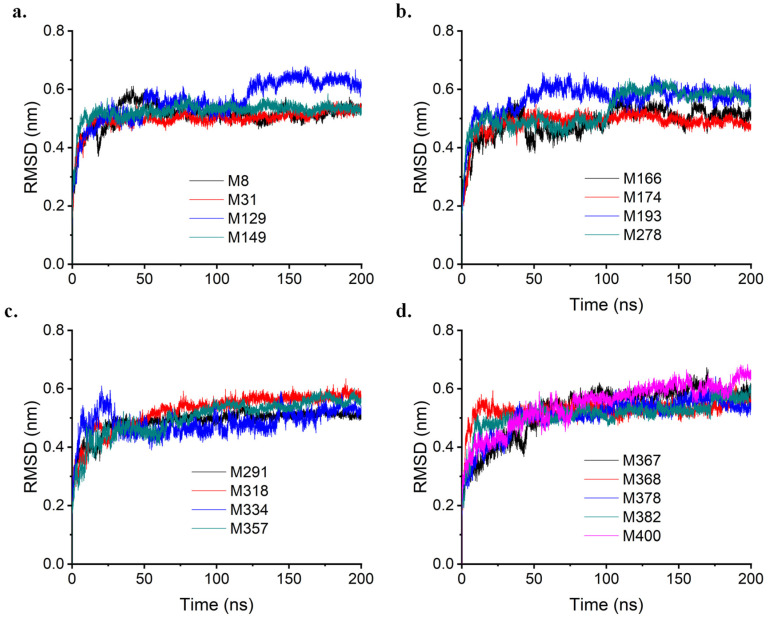
RMSD of Cytochrome B in the studied ligands during the 200-ns simulation (**a**–**d**).

Over the 200-nanosecond simulations (Figure 6), Cytochrome B displayed stable behavior across all studied systems, with maximum RMSD values ranging between 0.548 nm and 0.755 nm (Table A11). In most of the systems, the equilibrium is reached within the first 20 ns. However, while the complexes with Stigmatellin A and M235 maintained consistent structural integrity throughout the simulation, the systems involving M5 and M29 exhibited noticeable deviations after the 100-ns mark. Specifically, the M5-bound complex showed increased fluctuations, with RMSD values rising to approximately 0.6 nm, suggesting a possible conformational rearrangement or transient destabilization. In contrast, the M29 system remained relatively stable but displayed subtle shifts that may reflect localized structural adjustments within the binding site (Figure 6 and Figure A14). This underscores the importance of extended simulation times to capture conformational changes and ligand adjustments.

Previous studies have documented similar behavior, where ligand-induced strain or suboptimal binding geometries lead to dynamic responses in peripheral helices and loop regions [20,21]. These observations suggest that the structural perturbations seen in the M5 and M29 systems may represent an adaptive mechanism of the protein to accommodate less favorable ligands, or alternatively, an early indication of reduced binding stability.

**Figure A15:**
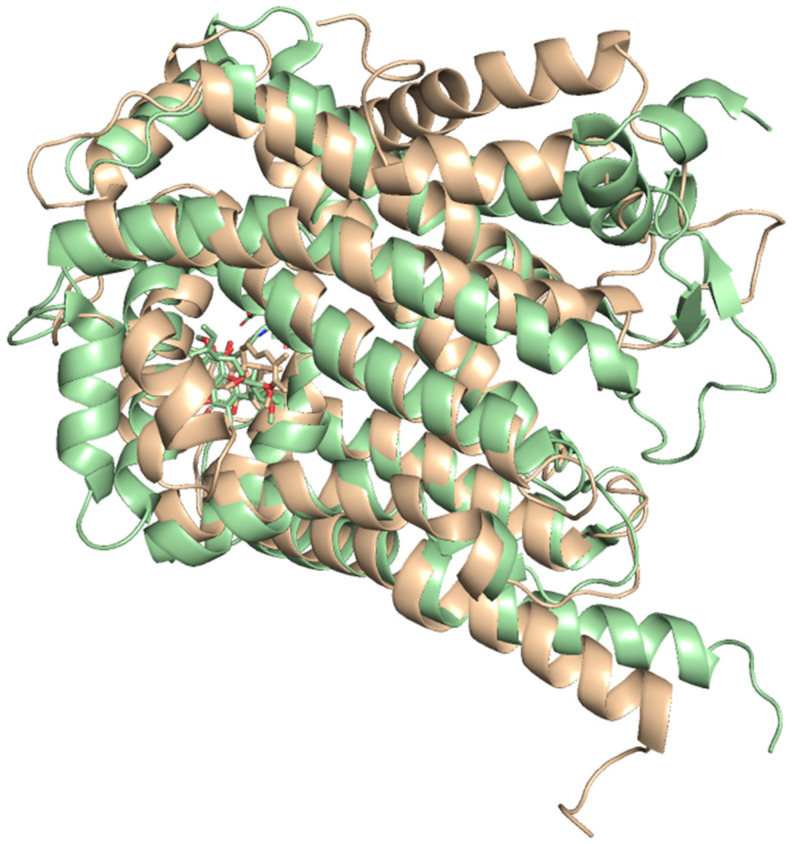
Crystal structure (pale green) and conformation after MD simulation (wheat) of Stigmatellin A-Cytochrome B complex superimposed to each other.

When comparing the crystal structure of Stigmatellin A–Cytochrome B complex to its conformation at the end of the simulation (reference system), an overall RMSD of 0.241 nm was observed. Key secondary structures near the active site remained well-aligned, while deviations were more pronounced in peripheral α-helices and loop regions. For the loops, this reflects their high flexibility, while for the peripheral α-helices, this phenomenon may be due to the absence of the other subunits within the full BC1 complex that may help stabilize them (Figure A15).

**Figure A16:**
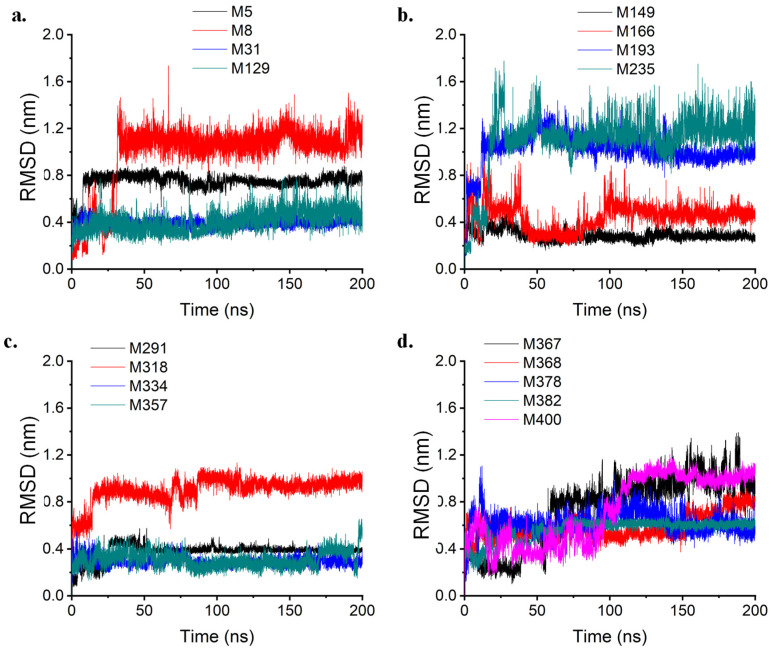
RMSD of Cytochrome B in the studied ligands during the 200-ns simulation (**a**–**d**).

Ligand dynamics were also evaluated via RMSD analysis (Figure 6 and Figure A16). As expected, ligand RMSD values were generally higher than those of the protein. Most ligands stabilized rapidly, often taking less than 5 ns and remained stable throughout the simulation. A few ligands required 50 to 80 ns to reach equilibrium, and others exhibited persistent fluctuations. Maximum RMSD values varied from 0.42 nm (Stigmatellin A) to 2.30 nm (M29). Notably, higher RMSD values did not always indicate instability. For instance, M174 maintained a high but consistent RMSD, indicating that the docking conformation found was not stable over time. Among the most stable ligands, Stigmatellin A had the lowest fluctuation (0.023 nm), followed by M31 and M334. The most unstable ligands included M29, M8, and M367 (Table A12).

During the 200-nanosecond simulation, the RMSD (Root Mean Square Deviation) profiles of the ligand–Cytochrome B complexes showed clear differences in how stable each structure was (Figure 6). As expected, the reference compound, Stigmatellin A, remained very stable throughout, with RMSD values consistently below 0.5 nm—indicating that it stayed firmly bound in its position. M278 behaved similarly, showing low and steady RMSD values, which suggests it fit well into the binding site and did not cause much structural disturbance.

On the other hand, M174 showed a slow and steady increase in RMSD during the first half of the simulation (about 100 ns), eventually leveling off around 1.25 nm. This likely means the molecule was gradually adjusting its shape before settling into a more comfortable position. M29 was the most unstable of the group. Its RMSD spiked quickly within the first 50 ns and kept fluctuating between 1.5 and 2.0 nm, which points to a poor fit in the binding site and ongoing structural shifts or temporary detachment.

**Figure 7:**
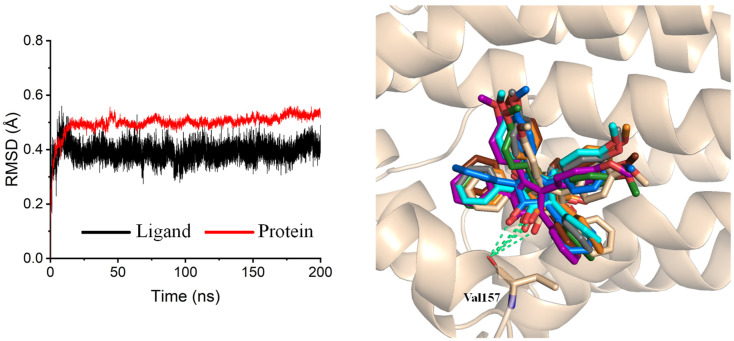
RMSD of the ligand and the protein in the M31-Cytochrome B system (**left**). Snapshot every 25 ns (**right**).

Interestingly, the behaviour of M31 contradicted expectations. Despite its poor initial docking score and unideal binding pose at the active site entrance, the ligand maintained a highly stable conformation throughout the entire 200 ns simulation. Snapshot analyses every 25 ns (Figure 7) confirmed that M31 remained consistently anchored near the entrance of the pocket. This unexpected stability suggests that M31 might exert a competitive inhibitory effect by blocking substrate access. Hydrogen bond analysis revealed a single bond formed between M31 and the backbone carbonyl of Val157, reinforcing the notion that hydrophobic interactions are the dominant binding force.

**Figure 8:**
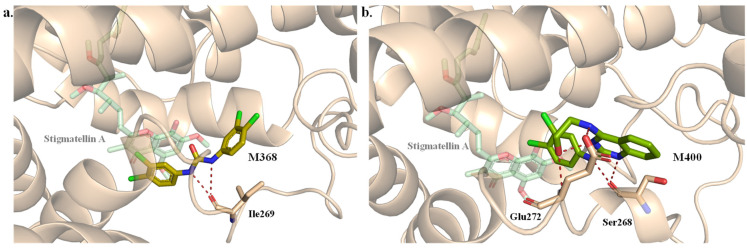
Interaction between M368 (**a**) and M400 (**b**) with Cytochrome B after 200 ns simulation. Stigmatellin A is shown in green transparency.

A broader hydrogen bond (HB) analysis showed that most systems maintained an average of 0 HBs during the simulation, with only M368 and M400 averaging 0.5 and 1.5 HBs, respectively (Table A13). Maximum HB formed ranged between 1 and 2 for most systems, while M368 and M400 formed 3 and 4 HBs, respectively. In M368, two nitrogen atoms and one oxygen enabled HB formation with the main chain carbonyl of Ile269 (Figure 8a). M400, on the other hand, shifted into a nearby sub-pocket during the simulation and formed stable HBs with the main chain of Ser268 and both the main and side chains of Glu272 (Figure 8b). Notably, this last interaction shows the potential relevance of the HB with Glu272 as observed previously in Stigmatellin A.

**Figure A17:**
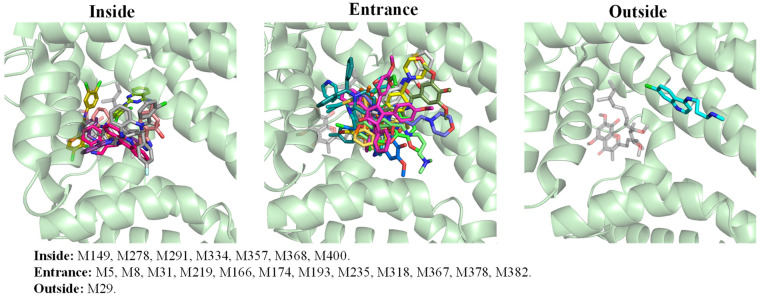
Final conformation of the studied ligands after the 200 ns simulation.

After completing the simulations, the conformational behavior of each ligand within the active site was analyzed. The results showed that seven compounds remained stably positioned inside the active site, indicating strong and consistent interactions. Twelve ligands, however, shifted toward the entrance of the active site, suggesting a less optimal fit or weaker binding affinity. Notably, one compound exited the active site entirely during the simulation, which points to a significantly weaker interaction. Interestingly, even after leaving the active site, this molecule maintained contact with a nearby α-helix, implying that some residual interaction persisted despite its displacement (Figure A17). This behavior suggests the presence of residual interactions that may not contribute to canonical binding but could still influence the protein’s conformational landscape. Such peripheral contacts are increasingly recognized as non-canonical interaction sites that can modulate protein dynamics and function [22]. Interestingly, the peripheral interactions have been previously reported in cytochrome systems, particularly in cytochrome P450 enzymes, where ligands interact transiently with residues lining access channels or flexible structural elements like helices and loops. These interactions can serve as transitional anchoring points or even contribute to allosteric modulation, subtly altering the protein’s conformational landscape and potentially affecting its functional state [23,24].

Lastly [25], the MM/PBSA binding free energy calculations confirmed that all ligand–Cytochrome bc1 complexes exhibited favorable interactions, as evidenced by consistently negative total ΔG values (Table 6). These results reinforce the findings from molecular docking and dynamics simulations, providing a thermodynamic perspective on ligand stability and affinity.

Stigmatellin A, used as the reference compound, showed the most favorable binding energy (ΔTOTAL = −54.99 kcal/mol), driven by strong van der Waals (ΔVDWAALS = −60.81 kcal/mol) and electrostatic (ΔEEL = −16.02 kcal/mol) contributions. Although the solvation energy (ΔGSOLV = +21.85 kcal/mol) partially offset these interactions, the overall binding remained highly favorable. This energetic profile aligns with its known potency as a Cytochrome B inhibitor and its stable behavior throughout the 200-ns simulation. The strong gas-phase interactions (ΔGGAS = −76.83 kcal/mol) suggest a tight and specific fit within the hydrophobic binding pocket, supported by hydrogen bonding with Glu272.

In contrast, M29 exhibited one of the least favorable binding energies (ΔTOTAL = −26.82 kcal/mol). While its van der Waals (−32.59 kcal/mol) and electrostatic (−1.26 kcal/mol) contributions were present, they were significantly weaker than those of Stigmatellin A. Moreover, the solvation energy (ΔGSOLV = +7.03 kcal/mol) further reduced the overall binding affinity. This thermodynamic profile is consistent with the ligand’s poor stability observed during MD simulations, where it showed high RMSD fluctuations and transient disengagement from the active site. The relatively low ΔGGAS (−33.85 kcal/mol) suggests suboptimal interactions within the binding pocket, likely due to poor shape complementarity or lack of key stabilizing contacts.

Interestingly, M278, which also demonstrated high structural stability during MD simulations, showed a highly favorable binding energy (ΔTOTAL = −51.42 kcal/mol). Its strong van der Waals (−60.78 kcal/mol) and electrostatic (−6.65 kcal/mol) interactions were partially offset by solvation effects (+16.01 kcal/mol), but the overall profile supports its potential as a competitive inhibitor. The similarity in binding orientation to Stigmatellin A, including π–π stacking with Tyr279, likely contributes to its strong gas-phase interactions (ΔGGAS = −67.43 kcal/mol).

Another notable case is M31, which, despite a poor docking score, exhibited a surprisingly favorable binding energy (ΔTOTAL = −40.84 kcal/mol). This was primarily due to a strong van der Waals contribution (−50.33 kcal/mol) and a relatively low solvation penalty (+9.81 kcal/mol). The ligand maintained stable contact with the entrance of the binding pocket throughout the simulation, suggesting a possible mechanism of competitive inhibition by blocking substrate access rather than deep binding.

Generally, the binding energy decomposition reveals that van der Waals interactions are the dominant stabilizing force across most systems, especially for ligands that fit well into the hydrophobic pocket of Cytochrome B. Electrostatic interactions, while variable, contribute meaningfully when hydrogen bonding or ionic contacts are present. Solvation energies tend to oppose binding but are outweighed in strong binders by favorable gas-phase interactions.

These findings underscore the importance of balancing hydrophobic complementarity, electrostatic interactions, and solvation effects in ligand design. Compounds like Stigmatellin A and M278 exemplify optimal profiles, while M29 highlights the limitations of poor fit and weak interactions. The MM/PBSA results thus provide a valuable thermodynamic validation of the structural and dynamic observations, guiding future optimization of antimalarial candidates targeting Cytochrome B.

## 3. Materials and Methods

### 3.1. Database Construction

The modeling dataset was derived from the Malaria Box collection, originally comprising 400 compounds with reported antimalarial activity. To ensure consistency and relevance, only those molecules with EC_50_ values determined against *Plasmodium falciparum* 3D7 strain and curated in the ChEMBL database were retained, resulting in a final set of 317 compounds. The structural diversity within this subset supports the development of predictive models with a broad applicability domain, enhancing their potential for generalization across chemically heterogeneous datasets.

Initially, canonical SMILES representations for the 317 selected compounds were retrieved. These were used to generate both two-dimensional and three-dimensional molecular structures via Open Babel. The 3D geometries were subsequently optimized using RDKit, applying molecular mechanics with the Universal Force Field (UFF) to ensure energetically favorable conformations. From these structures, three categories of molecular descriptors were computed: two-dimensional topological (2Dt), three-dimensional topological (3Dt), and quantum mechanical (QM), providing a comprehensive characterization of each molecule’s physicochemical and spatial properties.

2Dt descriptors were calculated with ToMoCoMD QuBiLs-MAS software v2.0, while 3Dt descriptors were calculated with ToMoCoMD QuBiLs-MIDAS [26]. The structures were optimized again at a semi-empirical level with the PM6 method, using Gaussian 16, to calculate the QM descriptors. From the Gaussian output files, the following descriptors were extracted: internal energy (U), enthalpy (H), Gibbs free energy (G), energy of highest occupied molecular orbital (E_HOMO), energy of molecular orbital lowest unoccupied (E_LUMO), polarizability, dipole moment (E_dipole_m), Log10 (Q), thermal energy (E), heat capacity (CV) and entropy (S). Additionally, the molecular weight, the number of hydrogen donors and the partition coefficient (AlogP) were added, whose values were reported in the Malaria Box. Finally, collinear descriptors were eliminated using a Spearman correlation coefficient and a Shannon entropy of 0.7, obtaining a total of 317 2Dt-QM descriptors and 499 3Dt-QM descriptors.

### 3.2. Construction of Regression Models

**Figure 9:**
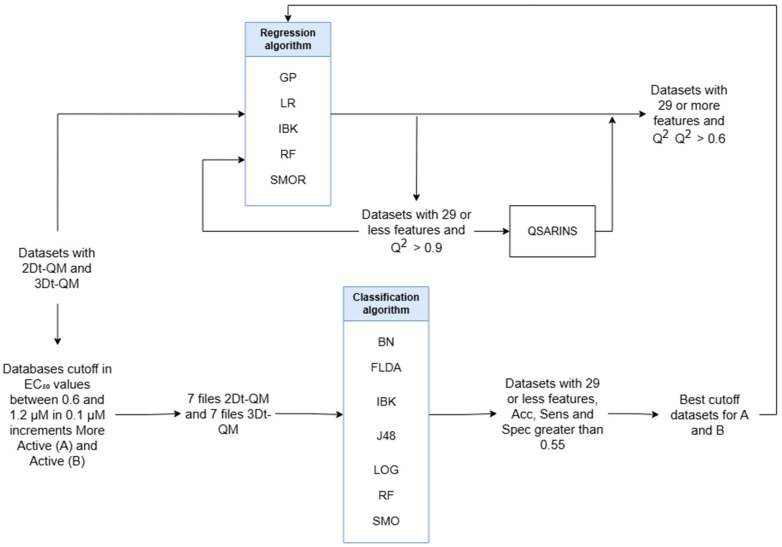
Block diagram for the feature selection of regression and classification models.

As an initial modeling strategy, regression analyses were conducted using two descriptor sets: 2Dt–QM and 3Dt–QM. Feature selection was performed using the wrapper method implemented in Weka 3.8, which, despite its computational intensity, is known to yield descriptor subsets with superior statistical performance compared to alternative techniques [14]. The regression models were built using a diverse set of algorithms, including Gaussian Processes (GP), instance-based learning with parameter k (IBK), multiple linear regression (LR), support vector regression (SMOR), and Random Forests (RF). Each algorithm was paired with one of three search strategies—Best First (BF), Greedy Stepwise (GS), and Genetic Algorithm (GEN)—to optimize descriptor selection and model performance. A schematic overview of this procedure is presented in Figure 9.

Finally, the 10-fold cross-validation coefficient (Q^2^_10-fold_) was analyzed as the main metric for the performance of the models. Those subsets with descriptors greater than 29 and a Q^2^_10-fold_ value close to 1 underwent a second search. This ensures that the ratio between the number of instances and the number of features is approximately 11:1, as suggested by the theoretical framework [14]. For modeling, the expression defined in Equation (1) is used as the dependent variable.

(1) $$pEC_{50}=-log(EC_{50},M)$$

### 3.3. Construction of Global Classification Models

Given the limited predictive performance observed in the initial regression models, a global classification strategy was adopted. The dataset was stratified into two categories, “very active” (A) and “active” (B), based on the EC_50_ values of each compound against Plasmodium falciparum. All compounds exhibited measurable biological activity; therefore, the classification was based on relative potency. Molecules with EC_50_ values equal to or below a defined threshold were assigned to class A, while those exceeding the threshold were placed in class B. To identify the optimal cutoff, a range of EC_50_ values between 0.6 and 1.2 µM was systematically evaluated in 0.1 µM increments. This process generated seven datasets for each descriptor type (2D-QM and 3D-QM), which were subsequently used to determine the most statistically robust partitioning and descriptor subsets for model development.

Subsequently, supervised feature selection was applied to each of the 14 datasets using the wrapper method, which is known for its ability to identify descriptor subsets with strong predictive relevance. To maintain model simplicity and avoid overfitting, descriptor sets containing more than 29 variables were excluded. A diverse array of classification algorithms was employed, including Bayes Network Learning (BN) [27], Fisher Linear Discriminant Analysis (FLDA) [28], Instance-Based Learning with parameter k (IBK) [29], Decision Trees based on information theory (J48) [30], Multinomial Logistic Regression with ridge regularization (LOG) [31], Random Forest (RF) [32] and Support Vector Classification using Sequential Minimal Optimization (SMO). For descriptor search optimization, Particle Swarm Optimization (PSO) and Incremental Wrapper Subset Selection (IWSS) were also incorporated, alongside the search strategies previously used in the global regression phase. A schematic overview of this procedure is presented in Figure 9.

To assess the performance of the classification models, a 10-fold cross-validation was conducted on all descriptor subsets that met the selection criteria. Models were developed using Weka 3.8, and their predictive capabilities were evaluated based on accuracy (Acc_10_-fold), sensitivity (Sens_10_-fold), and specificity (Spec_10_-fold). The most promising cutoff values and corresponding models were selected for further refinement. This entire process was automated through a Python 3 script utilizing the python-weka-wrapper3 library, ensuring reproducibility and efficiency. For external validation, the dataset was partitioned into training and test sets using the k-means clustering algorithm—a well-established method in predictive modeling literature. Clustering was performed in Minitab 20.3 17, using Euclidean distance as the similarity metric. Within each cluster, 75% of the compounds were randomly assigned to the training set, while the remaining 25% formed the test set, preserving structural diversity across both subsets.

To ensure the reliability of predictions, an applicability domain (AD) analysis was carried out using AMBIT Discovery. Four complementary methods were employed: nearest neighbor distance, Euclidean distance, range analysis, and probability density estimation. A compound was considered outside the model’s AD if more than two of these methods flagged it as such. Finally, model robustness was further evaluated using three validation strategies: 10-fold cross-validation on the training set, prediction on the external test set (Ext), and leave-one-out cross-validation (LOO). For each, key performance metrics were calculated, including accuracy, sensitivity, specificity, F-score, area under the ROC curve (AUC), and Matthews correlation coefficient (MCC).

Ensemble modeling was then initiated by calculating the prediction probabilities (P) for each compound across individual models. Based on these probabilities, ΔP values were computed—using Equation (2) for class A predictions and Equation (3) for class B. These ΔP values served as input features for selecting the most informative models to construct the ensemble. The ensemble’s performance was subsequently benchmarked against the best individual model using the same validation protocols.

(2) $$\Delta P_{A}=(1-2p)$$

(3) $$\Delta P_{B}=(2p-1)$$

### 3.4. Construction and Validation for Each Class Regression Model

After constructing the classification model, the dataset was split into two distinct groups, class A (very active) and class B (active), to enable targeted regression modeling for each class. Although the molecular instances differed between the two groups, the modeling workflow remained consistent. To begin, descriptor redundancy was addressed by removing collinear variables using a Spearman’s correlation threshold of 0.6 and a Shannon entropy cutoff of 0.7. This refinement yielded a total of 1185 3Dt–QM descriptors. The 2Dt–QM descriptors were excluded from this phase due to their previously observed poor performance in both regression and classification tasks.

Feature selection was then carried out using Weka 3.8.0, employing the same regression algorithms and search strategies used in the global modeling phase (see Figure 9). The quality of each model was assessed using the Q^2^_10_-fold metric, and the dataset was partitioned into training and test sets based on the classification-derived subsets to maintain consistency.

Model validation began with an applicability domain (AD) analysis, following the same protocol used in the classification stage. A compound was considered outside the AD if more than two of the four applied methods—nearest neighbor, Euclidean distance, range analysis, and probability density—flagged it as such.

To evaluate predictive performance and robustness, five statistical tests were conducted: correlation analysis, 10-fold cross-validation on the training set, external test set prediction (Ext), leave-one-out cross-validation (LOO), and y-randomization (y-scrambling). Key metrics such as the coefficient of determination (R^2^), Q^2^ values, mean absolute error (MAE), and root mean square error (RMSE) were calculated for each model.

Ensemble models were then constructed using the predictions obtained from the 10-fold cross-validation of individual models. A final round of feature selection was performed to identify the most informative models for ensemble integration. The resulting ensemble models were validated using the same statistical tests and benchmarked against the best-performing individual models to assess improvements in predictive accuracy and generalizability.

### 3.5. Molecular Docking

Molecular docking studies were conducted to explore the potential mechanisms of action of the selected antimalarial compounds. All 317 molecules from the Malaria Box database were used as ligands in the docking simulations. Additionally, Stigmatellin A, a well-characterized inhibitor of mitochondrial electron transport, was included as a reference compound to validate the docking protocol and provide comparative insights [25]. In this line, cytochrome BC1, a key component of the mitochondrial electron transport chain in Plasmodium falciparum, was selected as the target. This complex plays a central role in maintaining mitochondrial membrane potential and ATP synthesis, processes that are essential for parasite survival and proliferation [18,33]. In addition, cytochrome BC1 has been extensively validated as a drug target, particularly through the clinical success of atovaquone, a hydroxynaphthoquinone that inhibits the ubiquinol oxidation (Qo) site of the complex. On the other hand, cytochrome BC1 complex exhibits sufficient structural divergence between the parasite and human homologs, allowing for selective inhibition [34]. In this regard, differences in the active site, specifically at residues V259, P266, and F267 (K269, F276, and F277 in the human homologue), allow inhibitors to interact more tightly with the parasite variant than with the human one. This selectivity is crucial for providing a therapeutic window by minimizing host toxicity while maintaining potent antiparasitic activity. Recent research has also highlighted the Qi site of Cytochrome BC1 as a promising alternative target for next-generation quinolone-based antimalarials, which are being developed to overcome resistance mutations in the Qo site [21].

The X-ray diffraction crystal structure of *Saccharomyces cerevisiae* Cytochrome BC1 complex (PDB: 1KB9) was used as the receptor model [35]. Although direct crystallographic data for the parasite’s Cytochrome BC1 is limited, the yeast complex shares significant structural and functional conservation, particularly in the catalytic core subunits such as cytochrome b, cytochrome c1, and the Rieske iron–sulfur protein. In this context, the active site of *P. falciparum*, formed by F123, Y126, M133, W136, G137, A138, V140, I141, L144, I258, V259, P260, E261, F264, L265, P266, F267, Y268, and L271, is fully conserved in the *S. cerevisiae* homologue. This homology enables the use of yeast-derived models to study inhibitor binding and resistance mechanisms relevant to *P. falciparum* [34].

Receptor preparation was performed by deleting complex chains that were far and not in contact with the chain where inhibition happens, water molecules and other co-crystalized small molecules not involved in the inhibition mechanism were also deleted. Ligands and the receptor were prepared using AutodockTools v1.5.7, where polar hydrogens were added, and pdbqt format structures obtained [36]. For the calculations, AutoDock Vina was used, setting a 1 Å spacing, full ligand flexibility, and an exhaustiveness of 24 [37]. The grid box was reduced to the active site where Stigmatellin A binds (x = −12.801, y = 37.964, and z = −16.758) with a size of 20 Å for x, and 16 Å for y and z, respectively. Docking results were analysed, focusing on the docking score, pose, and hydrogen bonds formed.

### 3.6. Molecular Dynamics

Molecular dynamics (MD) simulations were used to evaluate the stability of the ligand-enzyme complex over time. As MD is a computationally expensive process, 20 ligands were randomly chosen (10 from the active ones and 10 from the most active ones). Stigmatellin A was also run as a reference. The output of the docking calculations was taken as the starting point for the MD. For the calculation, Gromacs 2019.6 was used. The topology of the Cytochrome was built using AMBER99SB-ILDN forcefield [38,39]. On the other hand, ligand topology was obtained using the Generalised Amber Force Field (GAFF) implemented in the ACPYPE server [40]. For the system solvation, a cube-shaped water box was used, combined with a three-point water model (TIP3) and neutralized using sodium or chlorine atoms as needed. Then, the system was relaxed and then equilibrated (100 ps at 300 K) using a NVT (constant number of particles, volume, and temperature) and NPT (constant number of particles, pressure, and temperature) protocols [41]. All simulations were run for a period of 200 ns, setting the pressure at 1 Barr and the temperature at 300 K. The MD run was first assessed by analyzing the volume, box size, temperature, density, pressure, and potential energy throughout the 200 ns, ensuring no erroneous behaviors occurred. Then, parameters such as the fluctuation, root-mean-square deviation, hydrogen bonds, and the stability of the ligand interaction with the receptor were studied.

(4) $$\Delta G_{binding}=G_{complex}-(G_{protein}+G_{ligand})$$

G_complex_ refers to the energy of the lead compounds-protein complex, while G_protein_ and G_ligand_ are denoted for the protein and ligand energy in water surrounded environment, respectively.

### 3.7. Binding Free Energy

The software gmx_MMPBSA was employed to calculate the binding free energies of protein−ligand complexes obtained for the molecular dynamics trajectory data, by applying the molecular mechanics/Poisson−Boltzmann (GeneralizedBorn) surface area (MM/PB(GB)SA) method [42,43]. The MD simulation trajectories from 0 to 200 ns were utilized to calculate the binding free energy of the complexes, considering a total of 200 frames. The representation of the binding free energy (ΔG_binding_) of the lead compounds in complex with protein was calculated using the following equation.

## 4. Conclusions

Ensemble models were constructed based on 2D and 3D topographic and quantum-mechanical (QM) molecular descriptors calculated at the semi-empirical level using the PM6 method. Initially, regression modeling was proposed to predict pEC_50_ values using 317 molecules from the Malaria Box database. However, the models built using both 2Dt-QM and 3Dt-QM descriptors showed Q^2^_10_-fold values below 0.7 and required a few descriptors beyond commonly accepted limits.

As a result, classification modeling was pursued by categorizing molecules into two classes, very active (A) and active (B), based on various EC_50_ cutoffs. Statistical evaluation using 10-fold cross-validation revealed that a cutoff of 0.7 provided the best classification performance. K-means clustering was employed to partition data for model validation, and applicability domain (AD) analysis using AMBIT Discovery confirmed 100% coverage for the training set—indicating broad predictive applicability across chemically diverse datasets.

An ensemble model (E_CLASS) was constructed using the best-performing classifiers, which outperformed the best individual model (M1_CLASS) in nearly all validation metrics. E_CLASS achieved an accuracy of 0.738, F-score of 0.742, AUC of 0.792, and a Matthews correlation coefficient of 0.477, confirming its superior predictive reliability.

Subsequently, regression modeling was separately applied to classes A and B, using attribute-selected 3Dt-QM descriptors and maintaining the same training/test partition. Ensemble models for both classes (E_REG_A and E_REG_B) significantly outperformed the best individual models. E_REG_A reached a Q^2^_10_-fold of 0.810 and Q^2^_ext_ = 0.749, while E_REG_B achieved a Q^2^_10_-fold of 0.793 and Q^2^_ext_ = 0.765. These results reinforce the advantages of ensemble strategies for predictive accuracy and generalizability. Descriptor analysis highlighted the importance of properties related to electronic distribution, molecular volume, and hydrophobicity, key parameters for both classification and regression modeling. These should be prioritized in future drug discovery studies targeting *P. falciparum*.

To further explore the potential mechanism of action, molecular docking and molecular dynamics simulations were performed using *P. falciparum* Cytochrome B, a validated antimalarial drug target. A reliable docking protocol was validated using Stigmatellin A, confirming that hydrophobic interactions and hydrogen bonding with Glu272 are critical for ligand binding. Of the 317 Malaria Box compounds, most displayed strong binding affinities, with docking scores ranging from −11.8 to −6.1 kcal/mol. Compounds with high docking scores or unstable poses were shown, via MD simulations, to be unlikely inhibitors of Cytochrome B. In contrast, compounds like M278 adopted conformations mimicking Stigmatellin A, forming stable interactions such as π–π stacking with Tyr279.

Interestingly, despite M31 showing an initially poor docking score, it showed an excellent conformational stability throughout a 200-ns simulation, hinting at a possible competitive inhibition mechanism by blocking the active site entrance. MD simulations of 20 representative compounds revealed that most ligands maintained stable binding over time, with hydrogen bond analysis highlighting Glu272, Ser268, and Ile269 as important interaction sites. Free binding energy calculations further validated the docking and MD findings, showing negative values for all ligands and confirming favorable binding profiles for many compounds.

Together, the integration of machine learning, docking, and molecular dynamics presents a comprehensive strategy for identifying, predicting, and characterizing novel antimalarial compounds.

## Figures and Tables

**Table 1:** The five best global regression models for an initial selection of features built with 2Dt-QM and 3Dt-QM, along with their statistical parameters for 10-fold cross-validation without the training/test partition (Q210-fold and MAE).

Model Name	Q^2^_10-fold_	MAE_10-fold_	Descriptor Type
GP_BF_67	0.363	0.266	2Dt-QM
GP_GS_77	0.360	0.268	
SMOR_BF_27	0.259	0.278	
SMOR_GS_22	0.259	0.275	
LR_BF_14	0.219	0.292	
GP_BF_144	0.792	0.159	3Dt-QM
GP_GS_144	0.770	0.168	
LR_BF_38	0.545	0.232	
LR_GS_38	0.545	0.232	
SMOR_BF_36	0.495	0.237	

**Table 2:** The best global classification models built with 2Dt-Q M and 3Dt-QM for an initial selection of features for each cutoff value in EC50, along with their statistical parameters for 10-fold cross-validation without the training/test partition (Acc_10-fold_, Sens_10-fold,_ and Sens_10-fold_).

Model Name	Cutoff	Acc_10-fold_	Sens_10-fold_	Spec_10-fold_	Descriptor Type
J48_GS_17	0.6	0.710	0.450	0.888	2Dt-QM
IBK_IWSS_17	0.7	0.672	0.644	0.701	
IBK_BF_8	0.8	0.672	0.726	0.606	
RF_BF_8	0.9	0.722	0.855	0.516	
IBK_IWSS_8	1.0	0.729	0.892	0.394	
IBK_BF_8	1.1	0.804	0.988	0.213	
BN_GEN_6	1.2	0.845	1.00	0.00	
SMO_BF_12	0.6	0.732	0.481	0.904	3Dt-QM
RF_BF_12	0.7	0.707	0.688	0.726	
SMO_BF_9	0.8	0.697	0.766	0.613	
IBK_BF_11	0.9	0.729	0.928	0.419	
IBK_IWSS_10	1.0	0.757	0.925	0.414	
LOG_BF_19	1.1	0.814	0.975	0.293	
IBK_BF_12	1.2	0.880	0.993	0.265	

**Table 3:** Statistical parameters for the validation of the best individual model (M1_CLASS) and the ensemble model (E_CLASS) with the training/test partition.

Parameter	M1_CLASS	E_CLASS
Acc_10-fold_	0.696	0.738
Sens_10-fold_	0.689	0.748
Spec_10-fold_	0.703	0.729
F Score_10-fold_	0.695	0.742
ROC_10-fold_	0.765	0.792
MCC_10-fold_	0.392	0.477
Acc_Ext_	0.675	0.675
Sens_Ext_	0.585	0.537
Spec_Ext_	0.769	0.821
F Score_Ext_	0.649	0.629
ROC_Ext_	0.690	0.708
MCC_Ext_	0.360	0.371
Acc_LOO_	0.717	0.734
Sens_LOO_	0.739	0.731
Spec_LOO_	0.695	0.737
F Score_LOO_	0.724	0.734
ROC_LOO_	0.779	0.789
MCC_LOO_	0.435	0.468

**Table 4:** The 5 best regression models for the classes: Very Active (A) and Active (B) built with 3Dt-QM, along with their statistical parameters (Q^2^_10-fold_ y MAE_10-fold_) for 10-fold cross-validation without the training/test partition.

Model Name	Q^2^_10-fold_	MAE_10-fold_	Class
LR_BF_70	0.944	0.079	A
LR_GS_71	0.944	0.079	
GP_BF_101	0.932	0.112	
GP_GS_131	0.931	0.114	
SMOR_BF_31	0.661	0.181	
GP_BF_112	0.930	0.046	B
GP_GS_117	0.922	0.048	
LR_BF_44	0.840	0.054	
LR_GS_44	0.840	0.054	
SMOR_BF_30	0.663	0.072	

**Table 5:** Statistical parameters for the validation of the best individual models and the ensemble models with the training/test partition for the classes: Very Active (A) and Active (B).

Parameter	M5_REG_A	E_REG_A	M1_REG_B	E_REG_B
R^2^	0.815	0.831	0.843	0.855
MAE	0.127	0.130	0.056	0.053
RMSE	0.184	0.177	0.069	0.067
Q^2^_10-fold_	0.729	0.810	0.708	0.793
MAE_10-fold_	0.174	0.139	0.075	0.065
RMSE_10-fold_	0.221	0.188	0.096	0.080
Q^2^_LOO_	0.775	0.802	0.725	0.804
MAE_LOO_	0.151	0.139	0.075	0.061
RMSE_LOO_	0.202	0.192	0.093	0.077
Q^2^_Ext_	0.621	0.749	0.681	0.765
MAE_Ext_	0.175	0.138	0.075	0.060
RMSE_Ext_	0.230	0.193	0.093	0.076
Q^2^_yScrambling_	0.025	0.025	0.026	0.026

**Table 6:** Free binding energy calculations for the studied systems in kcal/mol.

Molecule Name	ΔVDWAALS	ΔEEL	ΔEGB	ΔESURF	ΔGGAS	ΔGSOLV	ΔTOTAL	pEC_50_
M5	−38.95	−1.82	13.42	−5.43	−40.77	7.99	−32.78	2.34
M8	−28.42	−6.90	12.41	−3.96	−35.32	8.44	−26.88	2.06
M29	−32.59	−1.26	11.37	−4.34	−33.85	7.03	−26.82	0.36
M31	−50.33	−0.32	16.62	−6.81	−50.65	9.81	−40.84	2.70
M129	−47.29	−3.65	16.15	−5.96	−50.94	10.20	−40.75	1.21
M149	−49.07	−8.48	23.44	−6.41	−57.55	17.02	−40.53	4.19
M166	−40.13	−4.01	16.26	−5.42	−44.15	10.84	−33.30	1.03
M174	−44.63	−7.74	19.41	−5.60	−52.36	13.81	−38.55	0.70
M193	−38.47	−3.08	11.51	−5.13	−41.56	6.38	−35.18	2.19
M235	−40.53	−1.53	9.84	−5.44	−42.06	4.40	−37.66	1.14
M278	−60.78	−6.65	24.18	−8.16	−67.43	16.01	−51.42	2.61
M291	−43.38	−3.20	15.88	−5.69	−46.58	10.19	−36.39	1.05
M318	−36.49	−2.85	13.01	−5.17	−39.35	7.84	−31.51	0.57
M334	−43.27	−2.72	17.42	−5.73	−45.99	11.69	−34.30	1.71
M357	−54.88	−5.23	18.79	−7.09	−60.12	11.70	−48.42	2.07
M367	−43.91	−2.56	16.23	−5.89	−46.46	10.35	−36.12	1.65
M368	−42.63	−2.47	12.98	−4.93	−45.10	8.06	−37.04	1.72
M378	−34.60	2.51	10.86	−4.70	−32.09	6.16	−25.93	1.51
M382	−49.92	−0.47	11.23	−6.68	−50.39	4.55	−45.84	1.02
M400	−43.78	−12.27	22.23	−5.45	−56.05	16.79	−39.26	7.72
Stigmatellin A	−60.81	−16.02	29.81	−7.96	−76.83	21.85	−54.99	2.20

## Data Availability

The original contributions presented in this study are included in the article. Further inquiries can be directed to the corresponding authors.

## Abbreviations

The following abbreviations are used in this manuscript:

2Dt	Two dimensional topographical molecular descriptors
3Dt	Three dimensional topographical molecular descriptors
A	Very Active Class
AD	Applicability Domain
B	Active Class
CADD	Computer-aided drug design
CLASS	Classification
LOO	Leave one out cross validation
MAE	Mean Absolute Error
MCC	Mathews Correlation Coefficient
QSAR	Quantitative Structure-Activity Relationship
QM	Quantum Mechanical
REG	Regression
RMSE	Root Mean Squared Error
UFF	Universal Force Field

## Appendix A

**Table A1:** Summary of the 29 global regression models constructed with 2Dt-QM and 3Dt-QM descriptors, along with their statistical parameters for 10-fold cross-validation without the training/test partition (Q^2^_10-fold_ and MAE).

Model Name	Q^2^_10-fold_	MAE_10-fold_	Descriptor Type
GP_BF_67	0.363	0.266	2Dt-QM
GP_GS_77	0.360	0.268	
GP_GEN_123	0.122	0.326	
IBK_BF_6	0.201	0.292	
IBK_GS_6	0.201	0.292	
IBK_GEN_126	0.156	0.293	
LR_BF_14	0.219	0.292	
LR_GS_14	0.219	0.292	
LR_GEN_107	0.145	0.345	
SMOR_BF_27	0.259	0.278	
SMOR_GS_22	0.259	0.275	
SMOR_GEN_119	0.152	0.323	
RF_BF_4	0.180	0.294	
RF_GS_4	0.180	0.294	
RF_GEN_107	0.127	0.303	
GP_BF_144	0.792	0.159	3Dt-QM
GP_GS_144	0.770	0.168	
GP_GEN_205	0.353	0.294	
IBK_BF_9	0.228	0.286	
IBK_GEN_269	0.172	0.283	
LR_BF_38	0.545	0.232	
LR_GS_38	0.545	0.232	
LR_GEN_185	0.144	0.380	
SMOR_BF_36	0.495	0.237	
SMOR_GS_36	0.495	0.237	
SMOR_GEN_149	0.319	0.311	
RF_BF_15	0.245	0.293	
RF_GS_8	0.188	0.301	
RF_GEN_217	0.183	0.298	

**Table A2:** Summary of the 243 global classification models constructed with 2Dt-QM and 3Dt-QM descriptors for the seven EC50 cutoffs, along with their statistical parameters for 10-fold cross-validation without the training/test partition (Acc_10-fold_, Sens_10-fold_ and Spec_10-fold_).

Model Name	Cutoff	Acc_10-fold_	Sens_10-fold_	Spec_10-fold_	Descriptor Type
BN_BF_1	0.6	0.662	0.217	0.968	2Dt-QM
BN_GS_1		0.662	0.217	0.968	
BN_IWSS_1		0.662	0.217	0.968	
SMO_BF_7		0.707	0.372	0.936	
SMO_GS_7		0.707	0.372	0.936	
SMO_IWSS_17		0.694	0.395	0.899	
LOG_BF_4		0.688	0.349	0.920	
LOG_GS_4		0.688	0.349	0.920	
LOG_IWSS_11		0.653	0.395	0.830	
FLDA_BF_5		0.653	0.558	0.718	
FLDA_GS_3		0.637	0.535	0.707	
FLDA_IWSS_14		0.634	0.496	0.729	
IBK_BF_6		0.621	0.419	0.761	
IBK_GS_6		0.621	0.419	0.761	
IBK_IWSS_5		0.647	0.512	0.739	
J48_BF_19		0.700	0.434	0.883	
J48_GS_17		0.710	0.450	0.888	
J48_IWSS_4		0.678	0.287	0.947	
RF_BF_10		0.688	0.473	0.835	
RF_GS_7		0.678	0.419	0.856	
RF_IWSS_12		0.669	0.465	0.809	
BN_BF_6		0.644	0.186	0.957	3Dt-QM
BN_GS_6		0.644	0.186	0.957	
BN_IWSS_2		0.644	0.140	0.989	
SMO_BF_12		0.732	0.481	0.904	
SMO_GS_9		0.719	0.504	0.867	
SMO_IWSS_17		0.726	0.504	0.878	
LOG_BF_8		0.697	0.465	0.856	
LOG_GS_4		0.681	0.450	0.840	
LOG_IWSS_12		0.669	0.465	0.809	
FLDA_BF_7		0.700	0.651	0.734	
FLDA_GS_7		0.700	0.651	0.734	
FLDA_IWSS_13		0.669	0.643	0.686	
IBK_BF_2		0.672	0.535	0.766	
IBK_GS_2		0.672	0.535	0.766	
IBK_IWSS_6		0.644	0.457	0.771	
J48_BF_22		0.656	0.403	0.830	
J48_GS_16		0.666	0.388	0.856	
J48_IWSS_12		0.707	0.504	0.846	
RF_BF_10		0.710	0.496	0.856	
RF_GS_6		0.713	0.496	0.862	
RF_IWSS_22		0.700	0.488	0.846	
BN_BF_4	0.7	0.524	0.475	0.573	2Dt-QM
BN_GS_4		0.524	0.475	0.573	
BN_IWSS_1		0.517	0.569	0.465	
SMO_BF_10		0.628	0.588	0.669	
SMO_GS_8		0.606	0.575	0.637	
SMO_IWSS_19		0.644	0.475	0.815	
LOG_BF_3		0.599	0.631	0.567	
LOG_GS_3		0.599	0.631	0.567	
LOG_IWSS_14		0.596	0.613	0.580	
FLDA_BF_4		0.603	0.600	0.605	
FLDA_GS_2		0.612	0.625	0.599	
FLDA_IWSS_19		0.634	0.644	0.624	
IBK_BF_5		0.666	0.644	0.688	
IBK_GS_3		0.618	0.631	0.605	
IBK_IWSS_17		0.672	0.644	0.701	
J48_BF_16		0.615	0.575	0.656	
J48_GS_15		0.625	0.613	0.637	
J48_IWSS_2		0.568	0.169	0.975	
RF_BF_5		0.637	0.669	0.605	
RF_GS_5		0.637	0.669	0.605	
RF_IWSS_7		0.628	0.613	0.643	
BN_BF_4		0.552	0.156	0.955	3Dt-QM
BN_GS_4		0.552	0.156	0.955	
BN_IWSS_3		0.539	0.369	0.713	
SMO_BF_10		0.694	0.681	0.707	
SMO_GS_10		0.694	0.681	0.707	
SMO_IWSS_15		0.650	0.588	0.713	
LOG_BF_10		0.669	0.719	0.618	
LOG_GS_5		0.662	0.706	0.618	
LOG_IWSS_16		0.666	0.638	0.694	
FLDA_BF_6		0.612	0.606	0.618	
FLDA_GS_6		0.618	0.619	0.618	
FLDA_IWSS_17		0.653	0.631	0.675	
IBK_BF_2		0.644	0.694	0.592	
IBK_GS_2		0.644	0.694	0.592	
IBK_IWSS_13		0.653	0.650	0.656	
J48_BF_18		0.681	0.706	0.656	
J48_GS_12		0.678	0.694	0.662	
J48_IWSS_16		0.678	0.531	0.828	
RF_BF_12		0.707	0.688	0.726	
RF_GS_1		0.631	0.638	0.624	
RF_IWSS_17		0.659	0.631	0.688	
BN_PSO_12	0.8	0.552	1.000	0.000	2Dt-QM
BN_GEN_6		0.552	1.000	0.000	
BN_IWSS_1		0.552	1.000	0.000	
SMO_BF_5		0.596	0.840	0.296	
SMO_GS_5		0.596	0.840	0.296	
SMO_IWSS_1		0.552	1.000	0.000	
LOG_BF_6		0.647	0.737	0.535	
LOG_GS_6		0.647	0.737	0.535	
LOG_IWSS_14		0.612	0.720	0.479	
FLDA_BF_11		0.644	0.646	0.641	
FLDA_GS_2		0.603	0.634	0.563	
FLDA_IWSS_20		0.558	0.549	0.570	
IBK_BF_8		0.672	0.726	0.606	
IBK_GS_2		0.659	0.863	0.409	
IBK_IWSS_10		0.647	0.737	0.535	
J48_BF_17		0.625	0.823	0.380	
J48_GS_8		0.640	0.903	0.317	
J48_IWSS_8		0.634	0.851	0.366	
RF_BF_6		0.666	0.749	0.563	
RF_GS_3		0.618	0.686	0.535	
RF_IWSS_10		0.647	0.726	0.549	
BN_IWSS_1		0.527	0.760	0.239	3Dt-QM
SMO_BF_9		0.697	0.766	0.613	
SMO_GS_9		0.697	0.766	0.613	
SMO_IWSS_18		0.625	0.726	0.500	
LOG_BF_11		0.653	0.794	0.479	
LOG_GS_5		0.625	0.806	0.401	
FLDA_BF_7		0.666	0.669	0.662	
FLDA_GS_7		0.666	0.669	0.662	
FLDA_IWSS_21		0.653	0.629	0.683	
IBK_BF_2		0.647	0.731	0.542	
IBK_GS_2		0.647	0.731	0.542	
IBK_IWSS_15		0.640	0.703	0.563	
J48_BF_23		0.647	0.749	0.521	
J48_GS_21		0.640	0.743	0.514	
J48_IWSS_17		0.546	0.594	0.486	
RF_BF_9		0.631	0.703	0.542	
RF_GS_2		0.621	0.674	0.556	
RF_IWSS_14		0.593	0.651	0.521	
BN_BF_1	0.9	0.609	0.995	0.008	2Dt-QM
BN_GS_1		0.609	0.995	0.008	
BN_PSO_6		0.609	0.995	0.008	
BN_GEN_6		0.609	0.995	0.008	
BN_IWSS_1		0.609	1.000	0.000	
SMO_IWSS_1		0.609	1.000	0.000	
LOG_BF_9		0.691	0.917	0.339	
LOG_GS_6		0.691	0.917	0.339	
LOG_IWSS_20		0.625	0.731	0.460	
FLDA_BF_5		0.631	0.643	0.613	
FLDA_GS_5		0.631	0.643	0.613	
FLDA_IWSS_12		0.662	0.637	0.702	
IBK_BF_3		0.707	0.725	0.677	
IBK_GS_3		0.707	0.725	0.677	
IBK_IWSS_11		0.669	0.865	0.363	
J48_BF_17		0.653	0.855	0.339	
J48_GS_15		0.647	0.860	0.315	
J48_IWSS_16		0.703	0.824	0.516	
RF_BF_8		0.722	0.855	0.516	
RF_GS_4		0.710	0.834	0.516	
BN_IWSS_1		0.609	1.000	0.000	3Dt-QM
SMO_IWSS_1		0.609	1.000	0.000	
LOG_BF_10		0.703	0.845	0.484	
LOG_GS_10		0.703	0.845	0.484	
LOG_IWSS_15		0.621	0.793	0.355	
FLDA_BF_7		0.659	0.658	0.661	
FLDA_IWSS_15		0.637	0.601	0.694	
IBK_BF_11		0.729	0.928	0.419	
IBK_GS_1		0.634	0.881	0.250	
IBK_IWSS_6		0.669	0.839	0.403	
J48_BF_21		0.615	0.824	0.290	
J48_GEN_139		0.539	0.565	0.500	
J48_IWSS_12		0.685	0.834	0.452	
RF_GS_6		0.716	0.845	0.516	
RF_IWSS_17		0.681	0.850	0.419	
BN_PSO_11	1	0.672	1.000	0.000	2Dt-QM
BN_GEN_6		0.672	1.000	0.000	
BN_IWSS_1		0.672	1.000	0.000	
SMO_IWSS_1		0.672	1.000	0.000	
LOG_BF_15		0.726	0.892	0.385	
LOG_GS_7		0.710	0.906	0.308	
LOG_IWSS_17		0.722	0.864	0.433	
FLDA_IWSS_10		0.662	0.676	0.635	
IBK_BF_10		0.726	0.944	0.279	
IBK_GS_5		0.719	0.920	0.308	
IBK_IWSS_8		0.729	0.892	0.394	
J48_BF_10		0.710	0.953	0.212	
J48_GS_10		0.710	0.953	0.212	
J48_PSO_27		0.656	0.967	0.019	
J48_IWSS_9		0.732	0.836	0.519	
RF_IWSS_15		0.691	0.878	0.308	
BN_IWSS_1		0.672	1.000	0.000	3Dt-QM
SMO_IWSS_1		0.672	1.000	0.000	
LOG_BF_12		0.726	0.948	0.269	
LOG_GS_6		0.713	0.958	0.212	
LOG_IWSS_13		0.707	0.892	0.327	
FLDA_IWSS_10		0.716	0.728	0.692	
IBK_BF_9		0.738	0.939	0.327	
IBK_GS_4		0.732	0.944	0.298	
IBK_IWSS_10		0.757	0.925	0.414	
J48_BF_13		0.681	0.977	0.077	
J48_GS_11		0.672	0.967	0.067	
J48_IWSS_10		0.675	0.897	0.221	
RF_IWSS_12		0.726	0.916	0.337	
BN_PSO_11	1.1	0.763	1.000	0.000	2Dt-QM
BN_GEN_6		0.763	1.000	0.000	
BN_IWSS_1		0.763	1.000	0.000	
SMO_IWSS_1		0.763	1.000	0.000	
LOG_BF_3		0.767	0.996	0.027	
LOG_GS_3		0.767	0.996	0.027	
LOG_PSO_6		0.760	0.996	0.000	
LOG_GEN_17		0.751	0.967	0.053	
LOG_IWSS_1		0.763	1.000	0.000	
FLDA_IWSS_20		0.653	0.694	0.520	
IBK_BF_8		0.804	0.988	0.213	
IBK_GS_1		0.767	0.971	0.107	
IBK_IWSS_8		0.754	0.963	0.080	
J48_BF_9		0.767	0.992	0.040	
J48_PSO_23		0.760	0.992	0.013	
J48_IWSS_1		0.763	1.000	0.000	
RF_BF_8		0.785	0.975	0.173	
RF_IWSS_13		0.751	0.950	0.107	
BN_IWSS_1		0.763	1.000	0.000	3Dt-QM
SMO_IWSS_1		0.763	1.000	0.000	
LOG_BF_19		0.814	0.975	0.293	
LOG_GS_5		0.792	0.988	0.160	
LOG_IWSS_12		0.773	0.963	0.160	
FLDA_IWSS_13		0.650	0.657	0.627	
IBK_BF_1		0.785	0.967	0.200	
IBK_GS_1		0.785	0.967	0.200	
IBK_IWSS_10		0.782	0.971	0.173	
J48_BF_14		0.763	0.992	0.027	
J48_GS_10		0.757	0.984	0.027	
J48_IWSS_1		0.763	1.000	0.000	
RF_IWSS_13		0.770	0.975	0.107	
BN_PSO_11	1.2	0.845	1.000	0.000	2Dt-QM
BN_GEN_6		0.845	1.000	0.000	
BN_IWSS_1		0.845	1.000	0.000	
SMO_PSO_11		0.845	1.000	0.000	
SMO_GEN_6		0.845	1.000	0.000	
SMO_IWSS_1		0.845	1.000	0.000	
LOG_PSO_17		0.845	0.996	0.020	
LOG_GEN_6		0.845	1.000	0.000	
LOG_IWSS_1		0.845	1.000	0.000	
FLDA_IWSS_16		0.691	0.709	0.592	
IBK_IWSS_10		0.861	0.985	0.184	
J48_PSO_29		0.845	1.000	0.000	
J48_IWSS_1		0.845	1.000	0.000	
RF_IWSS_7		0.836	0.978	0.061	
BN_IWSS_1		0.845	1.000	0.000	3Dt-QM
SMO_IWSS_1		0.845	1.000	0.000	
LOG_BF_8		0.871	1.000	0.163	
LOG_GS_2		0.845	1.000	0.000	
LOG_IWSS_1		0.845	1.000	0.000	
FDLA_IWSS_20		0.820	0.955	0.082	
IBK_BF_12		0.880	0.993	0.265	
IBK_GS_2		0.852	1.000	0.041	
IBK_IWSS_5		0.849	0.993	0.061	
J48_IWSS_1		0.845	1.000	0.000	
RF_IWSS_8		0.836	0.985	0.020	

**Table A3:** Summary of the nine best global classification models constructed with 3Dt-QM descriptors for the 0.7 cutoff in EC50, along with their statistical parameters for the training/test partition and validation.

Parameter	RF_BF_12	IBK_IWSS_10	J48_GS_12	RF_IWSS_12	J48_BF_18
Acc_10-fold_	0.696	0.595	0.587	0.595	0.591
Sens_10-fold_	0.689	0.756	0.513	0.605	0.487
Spec_10-fold_	0.703	0.432	0.661	0.585	0.695
F Score_10-fold_	0.695	0.652	0.555	0.600	0.545
ROC_10-fold_	0.765	0.599	0.590	0.633	0.590
MCC_10-fold_	0.392	0.199	0.176	0.190	0.186
Acc_Ext_	0.675	0.563	0.588	0.563	0.500
Sens_Ext_	0.585	0.805	0.585	0.561	0.561
Spec_Ext_	0.769	0.308	0.590	0.564	0.436
F Score_Ext_	0.649	0.653	0.593	0.568	0.535
ROC_Ext_	0.690	0.559	0.612	0.558	0.461
MCC_Ext_	0.360	0.130	0.175	0.125	−0.003
Acc_LOO_	0.717	0.633	0.608	0.574	0.603
Sens_LOO_	0.739	0.840	0.630	0.580	0.613
Spec_LOO_	0.695	0.424	0.585	0.568	0.593
F Score_LOO_	0.724	0.697	0.617	0.577	0.608
ROC_LOO_	0.779	0.621	0.631	0.625	0.604
MCC_LOO_	0.435	0.291	0.215	0.148	0.207
Parameter	RF_IWSS_17	SMO_BF_10	J48_IWSS_16	IBK_IWSS_8	
Acc_10-fold_	0.650	0.679	0.608	0.624	
Sens_10-fold_	0.605	0.613	0.546	0.706	
Spec_10-fold_	0.695	0.746	0.669	0.542	
F Score_10-fold_	0.634	0.658	0.583	0.654	
ROC_10-fold_	0.714	0.680	0.596	0.636	
MCC_10-fold_	0.301	0.362	0.217	0.252	
Acc_Ext_	0.575	0.650	0.575	0.563	
Sens_Ext_	0.415	0.659	0.634	0.537	
Spec_Ext_	0.744	0.641	0.513	0.590	
F Score_Ext_	0.500	0.659	0.605	0.557	
ROC_Ext_	0.633	0.650	0.616	0.580	
MCC_Ext_	0.167	0.300	0.148	0.126	
Acc_LOO_	0.692	0.692	0.620	0.620	
Sens_LOO_	0.672	0.622	0.706	0.697	
Spec_LOO_	0.712	0.763	0.534	0.542	
F_Measure_LOO	0.687	0.670	0.651	0.648	
ROC_LOO	0.723	0.692	0.586	0.628	
MCC_LOO	0.384	0.388	0.243	0.243	

**Table A4:** Results of the ANOVA analysis for the accuracy (Acc 10-fold) and the MCC 10-fold of the best individual classification model (M1_CLASS) and the ensemble model (E_CLASS) with 95% CIs.

Model Name	Acc_10-fold_	MCC_10-fold_
E_CLASS	0.738	0.477
E_CLASS	0.743	0.485
E_CLASS	0.730	0.460
E_CLASS	0.738	0.477
E_CLASS	0.743	0.485
E_CLASS	0.730	0.460
E_CLASS	0.734	0.468
E_CLASS	0.738	0.477
E_CLASS	0.734	0.468
E_CLASS	0.734	0.468
M1_CLASS	0.696	0.392
M1_CLASS	0.675	0.350
M1_CLASS	0.709	0.418
M1_CLASS	0.679	0.359
M1_CLASS	0.705	0.409
M1_CLASS	0.675	0.350
M1_CLASS	0.700	0.401
M1_CLASS	0.696	0.393
M1_CLASS	0.684	0.367
M1_CLASS	0.717	0.435

**Table A5:** Summary of the statistical parameters (Q^2^10-fold and MAE) for the 15 regression models constructed for classes A and B using 3Dt-QM descriptors for an initial feature selection without the training/test partition.

Model Name	Q^2^_10-fold_	MAE_10-fold_	Class
GP_BF_101	0.932	0.112	Very Active (A)
GP_GS_131	0.931	0.114	
IBK_BF_10	0.439	0.224	
IBK_GS_8	0.389	0.234	
LR_BF_70	0.944	0.079	
LR_GS_71	0.944	0.079	
SMOR_BF_31	0.661	0.181	
SMOR_GS_31	0.661	0.181	
RF_BF_19	0.459	0.247	
RF_GS_13	0.450	0.244	
GP_GEN_612	0.350	0.254	
IBK_GEN_489	0.191	0.271	
LR_GEN_603	0.428	0.232	
SMOR_GEN_573	0.393	0.246	
RF_GEN_485	0.104	0.291	
*Second search GP_BF_101*			
GP_BF_88	0.938	0.110	
GP_GS_101	0.932	0.112	
GP_GEN_67	0.771	0.160	
IBK_BF_9	0.363	0.235	
IBK_GS_5	0.311	0.251	
IBK_GEN_47	0.317	0.254	
LR_BF_53	0.896	0.105	
LR_GS_60	0.898	0.106	
LR_GEN_61	0.765	0.152	
SMOR_BF_7	0.422	0.229	
SMOR_GS_7	0.422	0.229	
SMOR_GEN_58	0.714	0.176	
RF_BF_9	0.386	0.252	
RF_GS_6	0.369	0.249	
RF_GEN_54	0.193	0.285	
QSARINS_20	0.716	0.161	
*Second search GP_GS_131*			
GP_BF_84	0.913	0.114	
GP_GS_131	0.931	0.114	
GP_GEN_78	0.778	0.159	
IBK_BF_17	0.453	0.228	
IBK_GS_8	0.317	0.252	
IBK_GEN_74	0.350	0.254	
LR_BF_50	0.899	0.103	
LR_GS_51	0.887	0.112	
LR_GEN_73	0.695	0.183	
SMOR_BF_29	0.753	0.151	
SMOR_GS_29	0.753	0.151	
SMOR_GEN_75	0.692	0.184	
RF_BF_6	0.386	0.247	
RF_GS_5	0.371	0.253	
RF_GEN_76	0.192	0.282	
QSARINS_20	0.700	0.176	
*Second search LR_BF_70*			
GP_BF_49	0.854	0.137	
GP_GS_53	0.861	0.135	
GP_GEN_42	0.742	0.166	
IBK_BF_11	0.395	0.242	
IBK_GS_7	0.291	0.256	
IBK_GEN_37	0.302	0.256	
LR_BF_47	0.908	0.101	
LR_GS_48	0.908	0.101	
LR_GEN_46	0.823	0.14	
SMOR_BF_22	0.671	0.176	
SMOR_GS_20	0.654	0.177	
SMOR_GEN_34	0.727	0.164	
RF_BF_15	0.337	0.259	
RF_GS_6	0.332	0.264	
RF_GEN_41	0.227	0.277	
QSARINS_20	0.724	0.170	
*Second search LR_GS_71*			
GP_BF_49	0.854	0.137	
GP_GS_53	0.861	0.135	
GP_GEN_46	0.752	0.167	
IBK_BF_11	0.395	0.242	
IBK_GS_7	0.291	0.256	
IBK_GEN_35	0.431	0.228	
LR_BF_68	0.944	0.079	
LR_GS_71	0.944	0.079	
LR_GEN_44	0.798	0.149	
SMOR_BF_22	0.671	0.176	
SMOR_GS_20	0.654	0.177	
SMOR_GEN_49	0.744	0.166	
RF_BF_15	0.337	0.259	
RF_GS_6	0.332	0.264	
RF_GEN_28	0.268	0.267	
QSARINS_20	0.724	0.170	
GP_BF_112	0.930	0.046	Active (B)
GP_GS_117	0.922	0.048	
IBK_BF_17	0.554	0.083	
IBK_GS_6	0.356	0.102	
LR_BF_44	0.840	0.054	
LR_GS_44	0.840	0.054	
SMOR_BF_30	0.663	0.072	
SMOR_GS_28	0.650	0.075	
RF_BF_7	0.260	0.103	
RF_GS_4	0.259	0.107	
GP_GEN_507	0.206	0.116	
IBK_GEN_412	0.158	0.108	
LR_GEN_419	0.318	0.111	
SMOR_GEN_546	0.223	0.119	
RF_GEN_327	0.020	0.118	
*Second search GP_BF_112*			
GP_BF_92	0.925	0.047	
GP_GS_100	0.919	0.048	
GP_GEN_72	0.756	0.069	
IBK_BF_10	0.291	0.108	
IBK_GS_2	0.104	0.118	
IBK_GEN_54	0.302	0.103	
LR_BF_28	0.735	0.070	
LR_GS_28	0.735	0.070	
LR_GEN_58	0.620	0.086	
SMOR_BF_23	0.535	0.086	
SMOR_GS_18	0.504	0.088	
SMOR_GEN_72	0.803	0.060	
RF_BF_11	0.335	0.102	
RF_GS_4	0.279	0.107	
RF_GEN_44	0.190	0.109	
QSARINS_20	0.674	0.079	
*Second search GP_GS_117*			
GP_BF_112	0.927	0.047	
GP_GS_117	0.922	0.048	
GP_GEN_77	0.775	0.066	
IBK_BF_8	0.295	0.104	
IBK_GEN_61	0.319	0.102	
LR_BF_33	0.749	0.069	
LR_GS_34	0.749	0.069	
LR_GEN_65	0.706	0.073	
SMOR_BF_21	0.561	0.086	
SMOR_GS_21	0.561	0.086	
SMOR_GEN_73	0.757	0.067	
RF_BF_16	0.374	0.101	
RF_GS_15	0.354	0.102	
RF_GEN_60	0.180	0.110	
QSARINS_20	0.674	0.079	
*Second search LR_BF_44*			
GP_BF_44	0.805	0.065	
GP_GS_42	0.797	0.066	
GP_GEN_27	0.693	0.077	
IBK_BF_8	0.370	0.101	
IBK_GS_9	0.334	0.100	
IBK_GEN_23	0.399	0.099	
LR_BF_44	0.840	0.054	
LR_GS_44	0.840	0.054	
LR_GEN_29	0.707	0.074	
SMOR_BF_27	0.720	0.068	
SMOR_GS_27	0.695	0.070	
SMOR_GEN_32	0.708	0.072	
RF_BF_6	0.303	0.109	
RF_GS_6	0.303	0.109	
RF_GEN_24	0.232	0.109	
QSARINS_20	0.687	0.078	
*Second search LR_GS_44*			
GP_BF_44	0.805	0.065	
GP_GS_42	0.797	0.066	
GP_GEN_27	0.693	0.077	
IBK_BF_8	0.37	0.101	
IBK_GS_9	0.334	0.1	
IBK_GEN_23	0.399	0.099	
LR_BF_44	0.84	0.054	
LR_GS_44	0.84	0.054	
LR_GEN_29	0.707	0.074	
SMOR_BF_27	0.72	0.068	
SMOR_GS_27	0.695	0.07	
SMOR_GEN_32	0.708	0.072	
RF_BF_6	0.303	0.109	
RF_GS_6	0.303	0.109	
RF_GEN_24	0.232	0.109	
QSARINS_20	0.687	0.078	

**Table A6:** Summary of the statistical parameters for the validation of the five best regression models for class A, constructed with 3Dt-QM descriptors.

Parameter	LR_BF_70_SMOR_BF_22	GP_GS_131_QSARINS_20
R^2^	0.780	0.808
MAE	0.154	0.144
RMSE	0.199	0.186
Q^2^_10-fold_	0.648	0.693
MAE_10-fold_	0.197	0.184
RMSE_10-fold_	0.254	0.237
Q^2^_LOO_	0.639	0.721
MAE_LOO_	0.203	0.173
RMSE_LOO_	0.258	0.224
Q^2^_Ext_	0.545	0.493
MAE_Ext_	0.204	0.199
RMSE_Ext_	0.267	0.262
Q^2^_yScrambling_	0.0251	0.0259
Parameter	LR_GS_71_QSARINS_20	GP_GS_131_SMOR_BF_29
R^2^	0.827	0.818
MAE	0.137	0.153
RMSE	0.176	0.199
Q^2^_10-fold_	0.719	0.666
MAE_10-fold_	0.171	0.193
RMSE_10-fold_	0.225	0.254
Q^2^_LOO_	0.724	0.699
MAE_LOO_	0.172	0.186
RMSE_LOO_	0.224	0.244
Q^2^_Ext_	0.638	0.547
MAE_Ext_	0.186	0.187
RMSE_Ext_	0.235	0.249
Q^2^_yScrambling_	0.0249	0.0237
Parameter	GP_BF_101_QSARINS_20	
R^2^	0.815	
MAE	0.127	
RMSE	0.184	
Q^2^_10-fold_	0.729	
MAE_10-fold_	0.174	
RMSE_10-fold_	0.221	
Q^2^_LOO_	0.775	
MAE_LOO_	0.151	
RMSE_LOO_	0.202	
Q^2^_Ext_	0.621	
MAE_Ext_	0.175	
RMSE_Ext_	0.230	
Q^2^_yScrambling_	0.0254	

**Table A7:** Summary of the statistical parameters for the validation of the four best regression models for class B, constructed with 3Dt-QM descriptors.

Parameter	GP_BF_112_LR_BF_28	LR_BF_44_QSARINS_20
R^2^	0.843	0.769
MAE	0.056	0.067
RMSE	0.069	0.084
Q^2^_10-fold_	0.708	0.655
MAE_10-fold_	0.075	0.083
RMSE_10-fold_	0.096	0.104
Q^2^_LOO_	0.725	0.658
MAE_LOO_	0.075	0.082
RMSE_LOO_	0.093	0.103
Q^2^_Ext_	0.681	0.726
MAE_Ext_	0.075	0.066
RMSE_Ext_	0.093	0.081
Q^2^_yScrambling_	0.026	0.026
Parameter	GP_BF_112_QSARINS_20	LR_BF_44_SMOR_BF_27
R^2^	0.782	0.777
MAE	0.066	0.065
RMSE	0.082	0.083
Q^2^_10-fold_	0.691	0.606
MAE_10-fold_	0.079	0.089
RMSE_10-fold_	0.097	0.113
Q^2^_LOO_	0.678	0.608
MAE_LOO_	0.081	0.086
RMSE_LOO_	0.100	0.112
Q^2^_Ext_	0.665	0.827
MAE_Ext_	0.077	0.050
RMSE_Ext_	0.092	0.068
Q^2^_yScrambling_	0.027	0.027

**Table A8:** Results of the ANOVA analysis for the Q^2^10-fold and the RMSE10-fold of the best individual regression model for class A (M5_REG_A) and the ensemble model (E_REG_A) with 95% CIs.

Model Name	Q^2^_10-fold_	RMSE_10-fold_
E_REG_A	0.810	0.188
E_REG_A	0.801	0.192
E_REG_A	0.807	0.188
E_REG_A	0.803	0.190
E_REG_A	0.806	0.189
E_REG_A	0.800	0.191
E_REG_A	0.803	0.190
E_REG_A	0.800	0.191
E_REG_A	0.799	0.193
E_REG_A	0.814	0.186
M5_REG_A	0.729	0.221
M5_REG_A	0.764	0.206
M5_REG_A	0.770	0.203
M5_REG_A	0.769	0.204
M5_REG_A	0.744	0.215
M5_REG_A	0.748	0.213
M5_REG_A	0.771	0.203
M5_REG_A	0.753	0.211
M5_REG_A	0.761	0.207
M5_REG_A	0.752	0.211

**Table A9:** Results of the ANOVA analysis for the Q^2^10-fold and the RMSE10-fold of the best individual regression model for class B (M1_REG_B) and the ensemble model (E_REG_B) with 95% CIs.

Model Name	Q^2^_10-fold_	RMSE_10-fold_
E_REG_B	0.793	0.080
E_REG_B	0.806	0.077
E_REG_B	0.793	0.080
E_REG_B	0.796	0.079
E_REG_B	0.800	0.078
E_REG_B	0.803	0.078
E_REG_B	0.803	0.078
E_REG_B	0.788	0.081
E_REG_B	0.806	0.077
E_REG_B	0.797	0.079
M1_REG_B	0.708	0.096
M1_REG_B	0.703	0.096
M1_REG_B	0.711	0.095
M1_REG_B	0.685	0.100
M1_REG_B	0.665	0.103
M1_REG_B	0.708	0.095
M1_REG_B	0.730	0.091
M1_REG_B	0.679	0.100
M1_REG_B	0.681	0.101
M1_REG_B	0.678	0.101

**Table A10:** Docking score of all the studied molecules.

Molecule Name	Smiles	Docking Score (kcal/mol)
M1	COc1ccccc1CNC(=O)CCn1c(=O)[nH]c2ccsc2c1=O	−7.6
M2	CN(C)c1ccc(cc1)C(O)(c1ccc(cc1)N(C)C)c1ccc(cc1)N(C)C	−7.0
M3	c12cc(c3ccc(C)cc3)nn1ccnc2SCC(=O)Nc1c(Cl)ccc(C(F)(F)F)c1	−10.2
M4	CCOC(=O)C1=C(N=c2sc(=Cc3cc(C)n(c3C)c3ccc(F)cc3)c(=O)n2[C@@H]1c1cccc(OC)c1OC)c1ccccc1	−7.1
M5	CCOC(=O)c1cnc2c(C)cc(C)cc2c1Nc1ccc(OC)c(OC)c1	−7.1
M6	n12nc(nc1nc(C)cc2Nc1cc(C)cc(C)c1)C(F)(F)F	−9.1
M7	CC[C@]1(C)C[C@@](CCNCc2ccccc2O)(CCO1)c1ccc(C)cc1	−8.0
M8	CCc1c(C)c(=N)c2ccccc2n1C	−6.4
M9	Cc1cc(C=C2SC(=Nc3ccccc3)N(C3CCCC3)C2=O)c(C)n1c1ccc(Cl)cc1	−9.1
M10	Cc1cccc(Nc2nc(N)c3ccccc3n2)c1	−8.2
M11	COc1cc(OC)c(cc1Cl)N(C)C(=O)c1cc2CS(=O)(=O)c3ccccc3c2s1	−8.7
M12	Cc1ccc(cc1)N1C(=O)[C@H]2[C@@H](C1=O)[C@]1(C(=O)[C@@]2(C(=C1c1ccccc1)c1ccccc1)c1ccccc1)c1ccccc1	−7.9
M13	COc1ccc(Br)cc1[C@@H]1Nc2ccc3ccccc3c2C2=C1C(=O)C[C@H](C2)c1ccccc1	−9.2
M14	CCc1ccc(Nc2cc(C)c3ccc(O)cc3n2)cc1	−9.1
M15	CCn1c(CN2CCN(Cc3ccccc3)CC2)nc2cc(NC(=O)c3cccs3)ccc12	−10.2
M16	C(F)(F)(F)c1ccc(cc1)C(=O)NC[C@@H](N1CCCCCC1)c1ccsc1	−8.7
M17	Fc1ccc2[nH]c3c(NCCCN4CCOCC4)ncnc3c2c1	−7.9
M18	CCCCCC(=O)N1[C@H](C2=C(O)C[C@H](CC2=Nc2ccccc12)c1ccc(OC)c(OC)c1)c1ccc(cc1)C(F)(F)F	−8.3
M19	CCCc1c(CC)c(=N)c2ccccc2n1C	−6.8
M20	Cc1cc(Nc2ccc(C)c(Cl)c2)n2nc(nc2n1)C(F)(F)F	−8.7
M21	Cc1cc(NCCN2CCCCC2)c2ccc3ccccc3c2n1	−9.0
M22	CCOC(=O)c1cn(CC)c2cc(N(C)CCc3ccc(OC)c(OC)c3)c(F)cc2c1=O	−7.9
M23	CC1CCN(Cc2ccc(NC(=O)c3cc4ccc5cccnc5c4[nH]3)cc2)CC1	−10.0
M24	CSc1ccccc1C(=O)Nc1nc(cs1)c1ccccn1	−8.0
M25	[O-][N+](=O)c1ccc(C=NNc2nc3ccccc3[nH]2)cc1	−8.4
M26	COc1ccc(cc1)c1nc(NC(=O)CS(=O)(=O)c2ccc(F)cc2)sc1C	−8.9
M27	COc1ccc(cc1N1C(=O)[C@@H]2[C@H](C1=O)[C@@]1(C(=O)[C@]2(C(=C1c1ccc(C)cc1)c1ccc(C)cc1)c1ccccc1)c1ccccc1)C(=O)C	2.8
M28	CN(C)CCCNc1c2ccccc2nc2ccccc12	−6.7
M29	CNCCCNc1ccnc2cc(Cl)ccc12	−6.1
M30	COc1cccc(NC(=O)[C@H](C(C)C)n2c(=O)[nH]c3ccccc3c2=O)c1	−8.9
M31	COc1ccc(cc1)C1=C(c2ccc(OC)cc2)[C@@]2([C@@H]3[C@@H](C(=O)N(C3=O)c3ccc(C)cc3)[C@]1(C2=O)c1ccccc1)c1ccccc1	3.6
M32	CSC1=NC(=Nc2c(C)cccc2C)[C@]2(CC[C@H](CC2)C(C)(C)C)N1c1ccc(Cl)cc1	−7.9
M33	c12cc(c3ccc(OC)cc3)nn1ccnc2SCC(=O)Nc1c(Cl)ccc(C(F)(F)F)c1	−9.9
M34	n12nc(C)c(c3ccc(OC)c(OC)c3)c1ncc(C#N)c2N	−8.3
M35	c12c(C[C@H](C(=O)O)N[C@H]1c1ccc(Cl)cc1Cl)c1c(cccc1)[nH]2	−9.0
M36	COc1ccccc1CNC(=O)CCn1c(=O)[nH]c2ccccc2c1=O	−8.6
M37	Oc1ccccc1CNC[C@@]12C[C@@H]3C[C@@H](C[C@@H](C3)C1)C2	−7.6
M38	COc1ccc(C=C2SC(=N)N(C2=O)c2ccccc2OC)cc1	−7.3
M39	CC(C)(C)c1ccc(O)c(CNC2CCCCC2)c1	−7.6
M40	CCOC(=O)c1c(NC(=O)c2ccccc2)sc2c(O)c(CN(CC)CC)ccc12	−7.4
M41	n12ncnc1nc(C)cc2Nc1ccc2ccccc2c1	−8.9
M42	COc1ccc(NC(=O)[C@H]2[C@H](N(CC(C)C)C(=O)c3ccccc23)c2cccs2)cc1Cl	−7.1
M43	CCOC(=O)c1c(C)n(c2ccc(Cl)cc2)c2ccc(OC(=O)C=Cc3ccccc3)cc12	−9.1
M44	CN1CCN(CC1)c1ccc(Nc2c3ccccc3nc3ccccc23)cc1	−9.5
M45	CN(C)c1ccc(C=Cc2nc(=O)c3ccccc3o2)cc1	−8.4
M46	c12c(ccnc2cc(cc1)Cl)NCCN(CC)CC	−6.5
M47	C[C@H]1CN(C[C@@H](C)O1)C(=O)c1sc2ccccc2c1Cl	−7.6
M48	CCCN(CCC)C[C@@H](O)COc1ccccc1C(=O)Nc1ccccc1	−7.5
M49	COc1ccc2nccc([C@@H](O)[C@H]3C[C@@]4(CCN3CC4)C=C)c2c1	−7.7
M50	CC[C@H](c1ccccc1)c1nn2c(nnc2s1)c1cccs1	−8.4
M51	C(c1ccccc1)n1c(N=Nc2c(Nc3ccccc3)ccc3ccccc23)nc2ccccc12	−8.8
M52	O[C@@H](CNC1CCCCC1)Cn1c2ccccc2c2ccccc12	−8.6
M53	Cc1cc(C)c2CN(Cc3ccccc3Cl)COc2c1	−8.7
M54	Cc1ccc(cc1)N1[C@H]2N(c3ccccc3[C@@H]1N(c1ccccc21)S(=O)(=O)c1ccc(C)cc1)S(=O)(=O)c1ccc(C)cc1	−5.3
M55	CC(C)CN1[C@@H]([C@H](C(=O)Nc2cc(C)on2)c2ccccc2C1=O)c1cccs1	−7.7
M56	c12c(ncnc1NCCN(CC)CC)c1c(ccc(Cl)c1)[nH]2	−7.1
M57	Cc1ccc(cc1)c1cc2nc3CCCCc3c(O)n2n1	−8.8
M58	COc1ccccc1N=C1C(=O)c2ccccc2C(=C1N1CCCCC1)O	−7.3
M59	COc1ccc2c(=O)cc(oc2c1)C(Cl)(Cl)Cl	−7.2
M60	CCCc1cc(O)c2c3c(CN(C)CC3)c(=O)oc2c1	−7.5
M61	c1(cnc2c(c1O)cc(cc2)CC)C(=O)OCC	−7.1
M62	COc1ccc(cc1)c1nc2cc(Cc3ccc4[nH]c(nc4c3)c3ccc(OC)cc3)ccc2[nH]1	−11.7
M63	CCN1CCN(Cc2ccc(NC(=O)c3cc4ccc5cccnc5c4[nH]3)cc2)CC1	−9.9
M64	COc1ccc(c(OC)c1)n1ccnc1SCC(=O)Nc1cc(C)ccc1C	−8.5
M65	CCCCCCOC(=O)C1=C(C)NC(=O)N[C@H]1c1ccccc1F	−6.8
M66	n12nc(nc1nc(C)cc2Nc1ccc(cc1)Cl)C(F)(F)F	−8.5
M67	COc1cccc(C=Cc2nc(=O)c3ccccc3o2)c1OC	−8.7
M68	CCOc1cc2CCN[C@@H](c3cc(OC)c(O)cc3Cl)c2cc1OCC	−7.8
M69	CCOC(=O)c1cn(CC)c2cc(N3CCCCC3)c(F)cc2c1=O	−7.9
M70	n1c(NCCO)c2c(cccc2)nc1Nc1ccc(C)c(C)c1	−8.2
M71	[C@]1(O)(CC(=O)c2ccc(OC)cc2O1)C(F)(F)C(F)(F)F	−7.9
M72	COc1ccc(NC(=O)[C@@H]2[C@H](N(CC(C)C)C(=O)c3ccccc23)c2cccs2)cc1	−7.8
M73	c12cc(c3ccccc3)nn1ccnc2SCC(=O)Nc1c(Cl)ccc(C(F)(F)F)c1	−9.9
M74	C1CN(CCO1)c1ccc(Nc2ccnc3cc4ccccc4cc23)cc1	−10.1
M75	CCOC(=O)c1ccc2nc(cc(O)c2c1)c1ccccc1	−8.6
M76	[n+]1(C)c2c(cccc2)nc2ccc(NCCO)cc12	−6.8
M77	COc1ccc(cc1Cl)C(=O)Nc1nonc1c1ccc(OC)c(OC)c1	−7.5
M78	Cc1ccc(Nc2nc(cs2)c2ccccn2)cc1	−7.7
M79	CCOc1ccc(cc1OCC)c1nonc1NC(=O)c1cccc(C)c1	−8.6
M80	Cc1ccc(cc1)c1cc2C(=O)c3ccccc3c2nn1	−9.4
M81	CCOc1cc(Cl)c(cc1OCC)[C@H](C)NC(=O)c1ccccc1	−7.7
M82	C[C@H]1CCCCN1CCCNC(=O)c1ccc2c(c1)sc1nc(cn21)c1ccc(F)cc1	−9.2
M83	CCn1c(nc2ccccc2c1=O)SCC(=O)Nc1cc(ccc1Cl)C(F)(F)F	−9.1
M85	Cc1ccc(Cn2c(nc3ccccc23)N2CCC(CC2)C(=O)NCc2cccs2)cc1	−9.1
M86	CCCCc1ccc(NS(=O)(=O)c2cc3CCN4C(=O)CCc(c2)c34)cc1	−9.8
M88	COc1ccc(cc1)[C@H]1Sc2ccccc2N=C2C1=C(O)c1ccccc21	−7.9
M91	FC(F)(F)c1cccc(CNC2CCN(CCc3ccccc3)CC2)c1	−9.8
M92	CC[C@@H](C)NCC(O)(c1ccc(Cl)cc1)c1ccc(Cl)cc1	−7.4
M93	COCCN([C@@H](C(=O)NCc1ccc(OC)cc1)c1ccc(cc1)C(C)C)C(=O)Cc1cccs1	−7.8
M95	CCOc1ccccc1CNC(=O)[C@@H]1[C@@H](N(CC(C)C)C(=O)c2ccccc12)c1cccs1	−8.4
M97	Brc1ccccc1C(=O)Nc1nc(cs1)c1ccccn1	−8.3
M98	CCOc1ccc(C=Cc2nc(=O)c3ccccc3o2)cc1	−8.3
M99	c1ccc(cc1)c1ccc(cc1)c1nc2ccccc2[nH]1	−9.2
M102	CCCCNCC(O)(c1ccc(Cl)cc1)c1ccc(Cl)cc1	−7.4
M103	Clc1ccc(cc1)C1(CN2CCC(CC2)NC(=O)CCc2occc2)CCC1	−9.1
M105	Clc1ccc(cc1)S(=O)(=O)N1CCC(CC1)C(=O)NCc1occc1	−8.7
M109	COc1cccc(NC(=O)Cn2c(C)c(C#N)c3ccccc23)c1	−8.4
M111	CC(=CCN1CCC(CC1)C(=O)Nc1ccc(cc1)c1nc2ccccc2[nH]1)C	−8.5
M114	COc1cccc(O)c1CN1CC[C@@]2(CCCN(Cc3ccc(F)cc3)C2)C1	−8.8
M116	CCN1CCN(CC1)c1cc(C)c2cc(NC(=O)c3ccc(OC)c(OC)c3)ccc2n1	−8.2
M119	O[C@@H](Cn1c(=N)n(CCN2CCCCC2)c2ccccc12)c1cccc(Br)c1	−8.5
M121	COc1ccc(CNC2CCN(CC2)c2ccc(NC(=O)c3cccc(Cl)c3)cc2)cc1OC	−10.2
M124	C[C@H](CCC(=C)C)N1CC[C@@H](CC1)N1CC[C@H](CC1)C(=O)NCCc1ccccc1	−9.2
M128	C[C@H](NCc1ccccc1)c1cc(Cl)ccc1O	−7.7
M129	Brc1ccc(cc1)c1oc(cc1)C(=O)Nc1ccc(CN2CCOCC2)cc1	−8.8
M130	COc1ccc(CCN2CCC(CN(C)Cc3cnn(c3)c3ccccc3)CC2)cc1	−9.7
M131	Oc1c(ccc2cccnc12)[C@@H](NC(=O)c1ccccc1)c1ccccc1	−8.4
M133	CN1C(N(C)c2ccccc12)c1ccc2OCOc2c1	−8.1
M134	Cn1c(=O)c2cc(sc2c2ccccc12)C(=O)NCCCN1CCCCCC1	−8.9
M135	Cc1ccc(Cl)cc1N1CCN(CCCNC(=O)C2CCN(CC2)c2nc3ccccc3[nH]2)CC1	−10.8
M136	COc1cccc(c1)c1nn(C)c2sc(cc12)C(=O)NCCN1CCN(CC1)c1ccccc1F	−10.3
M137	n1c(NCCO)c2c(cccc2)nc1Nc1cccc(OC)c1	−7.5
M138	CCOc1cc(Cl)c(cc1OCC)[C@@H](C)NC(=O)c1cccc(OC)c1	−8.1
M141	Cc1cc(C)c2nc(sc2c1)N1CCC(CC1)C(=O)NCCCN1CCC(CC1)N1CCCCC1	−10.5
M143	Fc1ccc(NC(=O)Nc2ccc(Cl)c(Cl)c2)cc1Cl	−8.5
M145	COc1ccc(cc1)N1CCN(CC1)[C@@H](CNC(=O)C1CCCCC1)c1ccc2OCOc2c1	−8.5
M146	Cc1ccc(CSCc2oc(cc2)C(=O)NC2CC2)cc1	−7.7
M147	CC(C)c1ccc(Cn2c(CCNC(=O)C)nc3ccccc23)cc1	−8.9
M148	CCOc1cc(Br)c(cc1OCC)[C@@H](C)NC(=O)c1cccc(OC)c1	−8.2
M149	Cc1ccc(c(O)c1)c1cc([nH]n1)C(=O)Nc1ccc(cc1)S(=O)(=O)N1CCCCC1	−9.1
M150	O=C(NC[C@@H]1Cc2cccc(c2O1)c1ccncc1)c1csc2CCCCc12	−9.5
M151	Cc1[nH]c(cc1C(=O)NC1CCN(Cc2ccccc2)CC1)c1ccc(F)cc1	−9.5
M155	CC(C)OCc1cc(CN2CCN(CC2)c2cccc(Cl)c2)c(O)c2ncccc12	−8.7
M156	CCCN(CCC)CCCNC(=O)c1cc2c(nn(C)c2s1)c1ccc(OC)c(OC)c1	−7.6
M157	CCN(CC)CCn1c(=N)n(C[C@H](O)c2ccc(Cl)c(Cl)c2)c2ccccc12	−7.8
M160	Oc1ccccc1CN1CC[C@@]2(CCCN(Cc3ccccc3F)C2)C1	−9.5
M161	Cc1nc2ccccc2c(O)c1CC=C	−7.0
M162	CN1CCN(CC1)c1c2CCCc2c(C#N)c2nc3ccccc3n12	−6.8
M163	c12c(cnn1c1cccc(C)c1)c(=O)[nH]c(n2)n1nc(C)cc1NC(=O)c1cc2ccccc2o1	−8.3
M164	c12c(ncnc1NCCN(CC)CC)c1c(ccc(Br)c1)[nH]2	−7.0
M165	Clc1cc(NC(=O)c2oc(Br)cc2)ccc1N1CCCCC1	−8.0
M166	O=C(Nc1ccc(cc1)N1CCN(Cc2ccccc2)CC1)c1cccs1	−8.6
M167	C[C@H]1C[C@@H](C)CN(C1)C(=O)c1ccc(Cl)cc1Cl	−8.0
M168	Oc1ccccc1CN1CCN(CC1)C(c1ccccc1)c1ccccc1	−8.3
M169	COc1cc(OC)cc(c1)C(=O)Nc1ccc(cc1)c1nc2ccccc2[nH]1	−8.1
M170	COc1cc2cc(C(=O)Nc3ccccc3)c(N)nc2cc1OC	−8.4
M171	COc1nc(Nc2ccc(cc2)C(C)C)nc(OC)n1	−7.7
M172	n1(C[C@H](CNCCOC)O)c2c(cc(Cl)cc2)c2c1ccc(c2)Cl	−7.0
M173	n1(nc(C)cc1NC(=O)c1cccc(C(F)(F)F)c1)c1scc(c2ccc3OCOc3c2)n1	−10.0
M174	c12c(ncnc1NCCCN1CCOCC1)c1c(ccc(Cl)c1)[nH]2	−7.7
M176	O[C@@H](CNC1CCCC1)Cn1c2ccccc2c2ccccc12	−8.2
M177	Clc1ccc2nc(cc(C(=O)NC3CCN(Cc4ccccc4)CC3)c2c1)c1ccncc1	−9.7
M178	Clc1cc(Br)ccc1c1oc(CNCC2CCNCC2)cc1	−8.5
M179	CCOc1ccc(cc1)C1N(C)c2ccccc2N1C	−7.5
M180	c1(OCC)c(OCC)cc(cc1Cl)CN[C@@H](C)[C@H](O)c1ccccc1	−7.7
M181	CCc1ccc(Nc2cc(C)nc3nc(C)nn23)cc1	−7.9
M182	n12c3c(cccc3)nc1c1c(cccc1)N[C@]2(C)c1cccc(OC)c1	−8.0
M183	n1(CCc2ccccc2F)c2c(nc3c(cccc3)n2)c(C(=O)NCC(C)C)c1N	−8.4
M184	n12c3c(cccc3)nc1c(C#N)c(C)cc2N1CCN(CC1)C1CCCC1	−8.0
M185	CCN(CC)S(=O)(=O)c1ccc(cc1)c1csc(Nc2ccc(C)c(C)c2)n1	−7.5
M186	CC(=O)Nc1nnc(SCc2c(C)cc(cc2C)C(C)(C)C)s1	−8.4
M189	CCN(Cc1ccccc1)C1CCN(Cc2cc(Br)ccc2O)CC1	−9.0
M190	c1(Cl)c(ccc(Cl)c1)OC[C@@H](O)CNC(C)(C)CC(C)(C)C	−7.2
M191	n1(C[C@@H](CNC[C@H]2CCCO2)O)c2c(cc(Cl)cc2)c2c1ccc(c2)Cl	−7.8
M192	c12nc(=O)n(C)c(=O)c1nc(nn2CC)c1ccc(C(=O)OC)cc1	−8.4
M193	COc1cc(SC)ccc1C(=O)N1CCC[C@H](C)C1	−7.1
M194	c1(cc(C)nc2ccc(OC)cc12)Nc1ccccc1O	−8.6
M195	Cc1onc(NC(=O)c2ccc(cc2)c2ccccc2)c1	−8.9
M197	Brc1ccc(cc1)S(=O)(=O)N1CCC(CC1)C(=O)NCc1occc1	−8.6
M198	Clc1ccc(C=Cc2nc(=O)c3ccccc3o2)cc1	−8.6
M199	CC[C@H](O)c1ccc(Br)cc1NC(=O)c1cccc(Cl)c1	−7.2
M200	O=C(Nc1cccc(c1)C(=O)Nc1cccc(c1)c1nc2ccccc2[nH]1)c1occc1	−9.8
M201	C12=NC(C)(Cc3cc(OC)c(OC)cc3C1=NNC2=O)C	−6.8
M202	n12c3c(cccc3)nc1c1c(cccc1)N[C@]2(C)c1ccccc1	−7.9
M203	n1c(NCc2ccccc2)c2c(cccc2)nc1Nc1ccc(cc1)OC	−8.6
M204	Cc1cc(Nc2cccc(F)c2)nc(NCc2ccccc2)n1	−8.8
M206	Clc1ccc2nc(cc(C(=O)NC3CCN(Cc4ccccc4)CC3)c2c1)c1cccnc1	−9.9
M211	c1(OCC)c(OC)cc(cc1Cl)CNC[C@H](O)c1ccccc1	−7.8
M212	COc1ccc2nc(C)cc(Nc3ccc(Cl)cc3)c2c1	−8.7
M213	CCCCc1c(C)nc2c(OC)cccc2c1O	−7.0
M214	[C@@]12(Nc3ccccc3c3n1c1c(cccc1)n3)C(=O)Nc1ccc(OC)cc21	−7.7
M215	CCOC(=O)[C@H]1CCN(CC1)C(=O)CN1[C@H](c2ccccc2C1=O)c1c[nH]c2ccccc12	−8.7
M216	Clc1ccc(Cl)c(c1)c1oc(cc1)C(=O)Nc1ccc(CN2CCOCC2)cc1	−9.8
M217	CCOc1cc2CCN[C@@H](c3cc(C)c(O)c(C)c3)c2cc1OCC	−7.5
M218	Cc1sc2ncnc(Sc3nc4ccccc4[nH]3)c2c1C	−7.6
M219	CC(C)c1ccc(CNC[C@H](O)c2ccccc2)cc1	−6.7
M220	C[C@@H]1CCCC[C@H]1NC[C@H](O)Cn1c(C)c(C)c2ccccc12	−8.1
M222	c1(cc(Br)ccc1O)CN1CCN(CC1)Cc1cccc(F)c1	−8.3
M223	c12c(c(=O)n(C)c(=O)n1C)n(Cc1cccc(OC)c1)c(Oc1ccccc1)n2	−7.2
M224	CN(C)CCNc1cc(nc2ccccc12)c1ccccc1	−7.5
M227	COc1ccc2nc(C)cc(Nc3cccc(O)c3)c2c1	−8.0
M228	Clc1ccc2c(ccnc2c1)N1CCCC1	−7.3
M231	n12c(cc(C(C)(C)C)n1)nc1CCN(Cc3ccccc3OCC)Cc1c2O	−8.6
M232	n1c(cc(C)nc1Nc1ccc(cc1)NC(=O)CCc1ccccc1)N1CCCC1	−9.2
M233	c12c(ncnc1N1CCN(CC1)C)c1c(ccc(Cl)c1)[nH]2	−7.5
M235	CN(C)CCCNCc1oc(cc1)c1ccc(F)c(Cl)c1	−7.4
M237	O[C@H](CNCc1ccccc1)Cn1c2ccccc2c2ccccc12	−8.5
M239	CCOc1cc(cc(Cl)c1O)[C@@H]1NCCc2cc(OCC)c(OCC)cc12	−7.1
M242	COc1ccc(cc1)C(=O)[C@@H]1C[C@H]1c1ccc(Cl)cc1	−8.6
M243	COc1ccc(CN2CCN(Cc3ccccc3)CC2)c(O)c1	−7.9
M244	COc1ccc(cc1)C(=O)N[C@@H](c1ccc(Cl)cc1Cl)c1cc(Cl)c2cccnc2c1O	−8.3
M246	CCCCCCn1c(=N)n(C[C@@H](O)COc2cccc(C)c2)c2ccccc12	−7.9
M247	CCOC(=O)c1cnc2ccc(Cl)cc2c1NCCCN(C)C	−6.5
M248	OC1=C2[C@@H](Sc3ccccc3N=C2c2ccccc12)c1ccc(Br)cc1	−7.1
M250	Fc1cccc(c1)[C@H]1Sc2ccccc2N=C2[C@@H]1C(=O)c1ccccc21	−8.3
M252	Brc1ccc(cc1)c1cc2C(=O)c3ccccc3c2nn1	−9.0
M253	Cc1nc2ccccc2c(O)c1CC=C(Cl)Cl	−7.3
M255	Cc1ccccc1OCCSc1nc2ccc(NC(=O)c3ccccc3)cc2s1	−9.5
M256	Brc1ccc2OCC(=Cc2c1)C(=O)NCCCN1CC[C@H](CC1)N1CCCCC1	−9.6
M257	COc1ccc(OC)c(c1)[C@@H]1C2=C(CC(C)(C)CC2=O)N(C2=C1C(=O)CC(C)(C)C2)c1ccc(Cl)c(Cl)c1	−7.6
M258	CC(C)(C)c1ccc2OCN(Cc3ccc(Cl)cc3)Cc2c1	−9.0
M264	CCOC(=O)C1=C(C)N=c2sc(=Cc3cc(Cl)ccc3O)c(=O)n2[C@@H]1c1ccc(OC)cc1	−6.5
M266	Cc1ccnc(N[C@@H](c2cccc(OCc3ccccc3)c2)c2ccc3cccnc3c2O)c1	−9.2
M267	CCCC[C@@](O)([C@H](CN1CCOCC1)c1ccc(Cl)cc1)c1ccc(Cl)cc1	−7.9
M269	CC(C)CCNC[C@H](O)Cn1c2ccccc2c2ccccc12	−8.2
M270	FC(F)(F)c1cccc(Nc2nc3ccccc3nc2Nc2cccc(c2)C(F)(F)F)c1	−10
M271	COc1ccc2[C@@H]3CC(=Nc4ccccc4N3C(=O)c2c1OC)c1ccc(C)cc1	−8.3
M272	CC1=C(CC(=O)c2cccc(c2)[N+](=O)[O-])C(=O)c2ccccc2C1=O	−9.1
M273	CCCCCCc1cc(O)c2c3c(CC[C@@H](C)C3)c(=O)oc2c1	−8.8
M274	O=C(Nc1ccc(cc1)[C@@]12C[C@@H]3C[C@@H](C[C@@](C3)(C1)c1ccccc1)C2)c1cccs1	−10.4
M275	Cc1ccc2nc(C)cc(Nc3cccc(c3)C(F)(F)F)c2c1	−9.2
M276	COc1ccc(cc1)c1cc(=O)oc2c3CN(Cc4ccc(F)cc4)COc3ccc12	−10.1
M277	COc1ccc(CCNC(=O)c2cc(ccc2Sc2ccc(F)cc2)S(=O)(=O)N2C[C@@H](C)C[C@@H](C)C2)cc1OC	−8.7
M278	Cc1ccc(cc1)[C@H]1C[C@@H](n2nc(cc2N1)C(=O)N1CCN(CC1)C(c1ccccc1)c1ccccc1)C(F)(F)F	−11.8
M279	CC1CCN(Cc2c(O)ccc3cc(c(=O)oc23)c2nc3ccccc3s2)CC1	−9.3
M280	Clc1ccc2oc(=O)cc(NC3CCN(CCCc4ccccc4)CC3)c2c1	−9.2
M281	CCOC(=O)c1cnc2c(C)cccc2c1Nc1ccc(cc1)C(=O)C	−8.1
M282	Cc1ccc2cc3c(N)c4c(C)ccc(C)c4nc3nc2c1	−9.3
M283	COc1cccc(c1)C(=O)N[C@H](c1ccc(C)cc1)c1cc(Cl)c2cccnc2c1O	−8.7
M284	CCOc1ccc(Nc2c3ccccc3nc3ccccc23)cc1	−8.4
M285	CCOc1ccc2OCN(Cc3ccc(Cl)cc3)Cc2c1	−7.8
M286	CCOC(=O)C1=C(C)N=c2sc(=Cc3ccccc3O)c(=O)n2[C@@H]1c1ccc(OC)c2ccccc12	−7.4
M287	COc1ccc(cc1)[C@@H]1[C@@H](C(=O)Nc2cccc(OC)c2)c2ccccc2C(=O)N1C1CCCCC1	−7.1
M288	COc1ccccc1N1C(=N)SC(=Cc2ccc(OCc3ccccc3F)cc2)C1=O	−8.9
M289	CCOc1ccc(cc1)n1cc(c2ccccc2)c2c(SCc3cc(C)ccc3C)ncnc12	−9.1
M290	CCCCOC(=O)c1ccc(Nc2cc(C)nc3ccc(OC)cc23)cc1	−8.8
M291	C(Cc1c[nH]c2ccccc12)Nc1c2ccccc2nc2ccccc12	−9.1
M292	CCCc1c(C)nc2ccc(Br)cc2c1O	−7.2
M293	Nc1ccc(cc1)c1nc2cc(Cc3ccc4[nH]c(nc4c3)c3ccc(N)cc3)ccc2[nH]1	−11.6
M294	C(CCCNc1c2CCCCc2nc2ccccc12)CCNc1c2CCCCc2nc2ccccc12	−9.6
M295	Cc1ccc(C)c(Cn2c(nc3ccccc23)N2CCC(CC2)C(=O)NCc2cccs2)c1	−9.5
M296	COc1ccc(cc1CN1CCN(CC1)c1ccc(F)cc1)[C@H]1NCCc2c1[nH]c1ccccc21	−9.5
M298	COc1cc(OC)cc(c1)N1C(=O)N(Cc2ccc(cc2)C(C)(C)C)c2ccccc2S1(=O)=O	−8.1
M299	Brc1cccc(c1)C(=O)Nc1ccc(cc1)c1nc2cc3ccccc3cc2[nH]1	−9.9
M301	CCCOCc1cc(CN2CCN(CC2)c2cccc(Cl)c2)c(O)c2ncccc12	−8.4
M302	O=C(NC1CCCCC1)[C@@H](N(CCc1ccccc1)C(=O)c1occc1)c1cccs1	−6.7
M303	COc1ccc(cc1)C(=O)N[C@@H](c1ccccc1Cl)c1cc(Cl)c2cccnc2c1O	−8.5
M305	Cc1ccccc1OCCSc1nc2ccc(NC(=O)c3cccs3)cc2s1	−8.7
M307	CCOc1c(Br)cc(cc1OC)[C@@H]1C(=CN(C)C=C1C(=O)OC)C(=O)OC	−5.8
M308	Cc1ccc(F)cc1NC(=O)c1cc(Cl)ccc1Nc1ccccc1	−9.4
M309	COc1cc(cc(OC)c1O)c1nc(c2ccc(Sc3ccccc3)cc2)c([nH]1)c1ccccc1	−8.9
M312	O[C@H](Cn1nnc2ccccc12)Cn1c2ccc(Br)cc2c2cc(Br)ccc12	−9.0
M314	CCCCCCc1cc2cc(c(=Nc3ccccc3C)oc2cc1O)c1nc2ccccc2[nH]1	−9.5
M315	Brc1ccc(Nc2nc3ccccc3nc2Nc2ccc(Br)cc2)cc1	−8.7
M317	Cc1cc(Nc2ccc(OCc3ccccc3)cc2)nc2cc(O)ccc12	−10.1
M318	c1ccc(cc1)C1=Nc2ccccc2N=C(c2ccccc2)c2ccccc12	−8.2
M319	Cc1cccc(c1)N1C(=O)[C@H]2[C@@H](C1=O)[C@@]1(C(=O)[C@]2(C(=C1c1ccc2OCOc2c1)c1ccc2OCOc2c1)c1ccccc1)c1ccccc1	−3.1
M320	Oc1ccc2ccccc2c1[C@@H](NC(=O)Cc1ccccc1)c1ccc(Cl)cc1	−7.4
M322	c12c(ccnc1cc1ccccc1c2)NCCN(CC)CC	−7.8
M323	c12c(ncn(CCCn3ccnc3)c1=N)Oc1c(ccc3ccccc13)[C@H]2c1ccc(OC)c(OC)c1	−8.3
M324	C1(=C(NC2CCCCC2)C(=O)c2ccccc2C1=O)N(C)N=O	−7.6
M325	O=C(NC1CCN(Cc2ccccc2)CC1)c1cc(cc(c1)C(=O)NC1CCN(Cc2ccccc2)CC1)C(=O)NC1CCN(Cc2ccccc2)CC1	−8.9
M326	CCOc1cc2CCN[C@@H](c3cc(Cl)ccc3O)c2cc1OCC	−7.5
M328	CCCCc1c(CCC)c(=N)c2ccccc2n1C	−6.7
M329	C[C@@H]1CC2=C(C[C@@H]1CNCCCN(C)C)C(C)(C)CCC2	−7.3
M330	C[C@H](Oc1ccc(NC(=O)c2cccs2)cc1)C(=O)c1ccc(Cl)cc1	−8.8
M331	c1(C(=O)OCC)c2cc(ccc2n(c2ccccc2)c1C)OC(=O)c1cc(OC)c(c(c1)OC)OC	−8.4
M333	c12c(ncn(CCCN(C)C)c1=N)Oc1c(ccc3ccccc13)[C@H]2c1ccc(OC)cc1	−8.0
M334	C(Nc1nc(Nc2ccccc2)nc2ccccc12)c1ccccc1	−9.3
M335	Cc1cc(=O)oc2c1ccc1oc(C(=O)c3ccc(C)cc3)c(C)c21	−10.1
M336	COc1ccc(C=C2SC(=NC2=O)Nc2ccc(F)cc2)c(OC)c1	−8.4
M337	CCCCCCc1cc2cc(c(=O)oc2cc1O)c1csc(C=Cc2ccccc2)n1	−9.9
M338	COc1ccc(C=C2SC(=NC2=O)Nc2ccccc2)cc1	−7.8
M339	COc1ccc(CNC(=O)c2cc3c(nc4ccccn4c3=O)n(CCc3ccccc3)c2=N)cc1	−9.5
M340	Cn1c(=O)n(C)c2cc(CNCCNc3ccnc4cc(Cl)ccc34)ccc12	−9.0
M341	CCOc1ccc2nc(C)cc(Nc3ccc(cc3)N(C)C)c2c1	−8.1
M342	CCOC(=O)C1=CN(C)C=C([C@H]1c1cc(Br)c(OCC)c(OC)c1)C(=O)OCC	−5.9
M343	c1(C#N)nc(Cc2cccc3ccccc23)oc1N1CCN(CC1)C(c1ccccc1)c1ccccc1	−10.8
M344	CCOc1ccc2nc(C)cc(Nc3ccc(Br)cc3)c2c1	−8.2
M345	COc1ccccc1C(=O)ON=C(N)Cc1cccc2ccccc12	−8.9
M346	COc1cc2CCN(C)[C@H]3Cc4ccc(Oc5cc(C[C@H]6N(C)CCc7cc(OC)c(OC)c(Oc1cc23)c67)ccc5O)cc4	−4.4
M349	n1c(N)c2c(cccc2)nc1Nc1cccc(F)c1	−8.0
M350	COc1cccc(Nc2nc(N)c3ccccc3n2)c1	−8.0
M351	c1([C@H](c2cccs2)N2CCN(c3ccccc3)CC2)c(C)c(C)sc1NC(=O)c1ccccc1	−7.1
M352	c1(Nc2ccc(cc2)N2CCN(CC2)C)c2c(ccc(OC)c2)ncc1C(=O)OCC	−8.2
M353	n12c(nc(c3ccc(Cl)s3)cc1C(F)(F)F)cc(C(=O)NC1CCN(CC1)Cc1ccccc1)n2	−9.0
M354	c1(N2CCN(CC2)C)c2c(nc(Nc3ccc(O)cc3)n1)cccc2	−6.9
M355	CN1C=C([C@@H](c2ccccc12)c1c[nH]c2ccccc12)c1ccc2ccccc2n1	−10.3
M356	Oc1ccc(cc1)c1nc(c2ccc3oc4ccc(cc4c3c2)c2[nH]c(nc2c2ccccc2)c2ccc(O)cc2)c([nH]1)c1ccccc1	−9.6
M357	CC(C)(C)c1ccc(cc1)[C@H]1CC(=C2[C@@H](N(C(=O)c3ccccc3Cl)c3ccccc3N=C2C1)c1ccc(F)cc1)O	−9.6
M358	O[C@@H](CNC1CCCCC1)Cn1c2ccc(I)cc2c2cc(I)ccc12	−8.7
M360	O=C(Nc1cccc(c1)C1=NCCN1)Nc1cccc(c1)C1=NCCN1	−9.3
M361	ClCC(=O)NCCOc1ccc(Cl)cc1Cl	−6.2
M363	c12c(cc(C(=O)NCc3cccnc3)c(=N)n1CCc1ccc(cc1)OC)c(=O)n1cccc(C)c1n2	−9.1
M364	n1c(Nc2ccc(cc2)OC)cc(C)nc1NCc1ccccc1	−8.8
M365	OCCCCn1c(=O)c2ccc3c(=O)n(CCCCO)c(=O)c4ccc(c1=O)c2c34	−7.4
M367	COc1cc(CNCCc2ccc(Cl)cc2)cc(OC)c1O	−7.4
M368	Clc1ccc(NC(=O)Nc2ccc(Cl)c(Cl)c2)cc1Cl	−8.4
M369	c12[nH]nc(c3c(O)cc(C)c(Cl)c3)c2[C@H](c2ccccc2F)N(C1=O)CCc1ccc(cc1)OC	−8.7
M370	n1(ccc(N)nc1=O)[C@H]1O[C@@H]([C@@H](O)[C@H]1O)CO	−5.5
M371	c12c(nc(c3ccc(OC)cc3)nc1N1CCN(C(=O)C3CCCCC3)CC1)sc1CCCCc21	−5.9
M372	c1(sc2cc3c4c(CC3)cccc4c2n1)NC(=O)c1cc(OC)c(c(c1)OC)OC	−8.3
M373	n1(CC(=O)c2ccc(Cl)c(Cl)c2)c2c(cccc2)n(CCN2CCCCC2)c1=N	−8.9
M375	Oc1ccc2ccccc2c1CNc1nc2ccccc2[nH]1	−9.3
M376	COc1cc(cc(OC)c1O)c1nc(c2ccccc2)c([nH]1)c1ccccc1	−8.0
M377	CCOc1ccc(CN2CCN(C[C@@H](O)COc3ccc(OC[C@H](O)CN4CCN(Cc5ccc(OCC)cc5)CC4)cc3)CC2)cc1	−8.8
M378	COc1cc(cc(OC)c1OC)C(=O)Nc1ccc(cc1)c1nc2ccccc2[nH]1	−7.9
M379	CCCCNc1nc(NC(C)(C)C)nc(NC(C)(C)C)n1	−5.8
M380	Oc1c(ccc2cccnc12)[C@@H](Nc1ccccn1)c1cccc(Oc2ccccc2)c1	−9.5
M381	CCOc1ccc(Nc2ccnc3cc(Cl)ccc23)cc1	−8.0
M382	c1([C@H](c2cccnc2)N2CC[C@@H](CC2)Cc2ccccc2)c(C)c(C)sc1NC(=O)c1ccco1	−7.1
M383	Cc1ccc2c(C)nc(Nc3nc(C)cc(n3)c3ccccc3)nc2c1	−7.9
M384	CCn1c2c(CCC2)c(=N)c2c1CCCC2	−6.8
M385	COc1cc(cc(OC)c1OC)C(=O)ON=C(N)Cc1cccc2ccccc12	−8.6
M386	C12=C(c3c(cccc3)C1=O)Nc1c(cccc1)S[C@@H]2c1ccccc1	−8.1
M387	COC(=O)c1ccc(cc1)[C@@H]1N(C(=O)c2ccccc2)c2ccccc2N=C2C[C@@H](CC(=C12)O)c1ccc(F)cc1	−8.0
M388	Clc1ccc(cc1Cl)C(=O)Nc1ccc(cc1)c1nc2ccccc2[nH]1	−9.0
M389	CC(=O)c1sc(NC(=O)Nc2ccc(C)cc2C)nc1C	−8.2
M390	Cc1ccc(Nc2nc(N)c3ccccc3n2)cc1Cl	−9.1
M391	n1c(N(C)C)cc(C)nc1Nc1ccc(cc1)NC(=O)Nc1ccccc1	−9.0
M392	N1[C@@H](c2cccc3ccccc23)[C@H]2CC=C[C@H]2c2c1c(Cl)ccc2C(=O)OC	−9.4
M393	c1(nc(cc(n1)NNC(=O)c1ccccc1)N1CCCC1)N1CCCC1	−7.8
M394	O[C@H](COc1ccccc1C(=O)CCc1ccccc1)CN1CCCCC1	−7.8
M395	CCCn1c(=N)c(cc2c1nc1n(cccc1C)c2=O)C(=O)NC1CCCCC1	−9.0
M396	Nc1ccc(cc1)c1nc2cc(Oc3ccc(Oc4ccc5[nH]c(nc5c4)c4ccc(N)cc4)cc3)ccc2[nH]1	−10.9
M397	COc1ccc(Nc2nc(NN=Cc3ccc(O)cc3)nc(Nc3ccc(cc3)[N+](=O)[O-])n2)cc1	−7.7
M400	OCCNc1nc(Nc2ccc(Cl)c(Cl)c2)nc2ccccc12	−7.9
Stigmatellin A	C/C=C(\C)/C=C/C=C/[C@@H]([C@@H](C)[C@H]([C@@H](C)CCC1=C(C(=O)C2=C(O1)C(=C(C=C2OC)OC)O)C)OC)OC	−9.1

**Table A11:** Max and average RMSD values of Cytocrhome B in each studied system after 200 ns simulation.

Molecule Name	Max RMSD (nm)	Average RMSD (nm)
M5	0.755	0.596
M8	0.611	0.510
M29	0.695	0.479
M31	0.561	0.498
M129	0.680	0.561
M149	0.587	0.524
M166	0.586	0.486
M174	0.548	0.485
M193	0.658	0.566
M235	0.569	0.506
M278	0.638	0.531
M291	0.567	0.488
M318	0.635	0.524
M334	0.610	0.483
M357	0.603	0.501
M367	0.673	0.528
M368	0.617	0.524
M378	0.642	0.505
M382	0.618	0.512
M400	0.684	0.544
Stigmatellin A	0.725	0.595

**Table A12:** Max, average RMSD, and RMSD fluctuation values of the ligands in each studied system after 200 ns simulation.

Molecule Name	Max RMSD (nm)	Average RMSD (nm)	RMSD Fluctuation (nm)
M5	0.900	0.747	0.046
M8	1.736	0.981	0.315
M29	2.301	1.067	0.619
M31	0.560	0.392	0.024
M129	0.814	0.403	0.071
M149	0.590	0.287	0.034
M166	1.081	0.445	0.086
M174	1.449	0.974	0.190
M193	1.439	1.023	0.090
M235	1.775	1.104	0.166
M278	0.498	0.375	0.038
M291	0.575	0.379	0.043
M318	1.133	0.906	0.076
M334	0.554	0.293	0.031
M357	0.654	0.310	0.055
M367	1.392	0.746	0.276
M368	0.915	0.587	0.091
M378	1.107	0.621	0.071
M382	0.685	0.563	0.080
M400	1.205	0.729	0.270
Stigmatellin A	0.420	0.254	0.023

**Table A13:** Maximum, minimum, and average HB between each ligand and cytochrome B during the 200 ns simulation.

Molecule Name	Max HB	Min HB	Average HB
M5	1	0	0.0
M8	2	0	0.0
M29	2	0	0.0
M31	1	0	0.0
M129	1	0	0.0
M149	2	0	0.0
M166	1	0	0.0
M174	1	0	0.0
M193	0	0	0.0
M235	1	0	0.0
M278	0	0	0.0
M291	2	0	0.0
M318	1	0	0.0
M334	1	0	0.0
M357	1	0	0.0
M367	2	0	0.1
M368	3	0	0.5
M378	1	0	0.0
M382	0	0	0.0
M400	4	0	1.5
Stigmatellin A	1	0	0.0
